# Beyond Known PFAS: Emerging Analytical Strategies for Resolving the Hidden Fluorinated Chemical Space

**DOI:** 10.3390/toxics14100924

**Published:** 2026-10-19

**Authors:** Faisal Ahmed, Elif Yakamercan, Mahmut S. Ersan

**Affiliations:** 1 Department of Civil Engineering, University of North Dakota, Grand Forks, ND 58202-8115, USA; 2 Department of Environmental Engineering, Bursa Technical University, Bursa 16310, Türkiye

**Keywords:** unknown PFAS, environmental occurrence, PFAS detection, extractable organofluorine, non-targeted analysis, adsorbable organofluorine, fluorine mass balance

## Abstract

Per- and polyfluoroalkyl substances (PFAS) occur as complex mixtures in natural and engineered systems, yet routine monitoring captures only a small fraction of the broader PFAS chemical space. Across waters, solids, and biota, measurements of total and extractable organofluorine frequently exceed the fluorine accounted for by targeted PFAS, revealing substantial unidentified organofluorine fractions that may contain unidentified PFAS, precursors, transformation products, and other fluorinated compounds. This review critically evaluates the occurrence, analytical characterization, transformation, and detection of unknown PFAS across environmental and engineered systems. We critically synthesize current knowledge on (i) the occurrence, sources, and major environmental reservoirs of unknown PFAS; (ii) complementary analytical strategies for constraining and identifying the unknown fraction, including high-resolution mass spectrometry (HRMS)-based suspect and non-target screening, ion mobility HRMS, organofluorine mass-balance approaches (EOF/AOF/TF), total oxidizable precursor (TOP) assays, Bayesian-assisted precursor inference, and fluorine-specific techniques; and (iii) current analytical uncertainties. Finally, we identify research priorities including standardized fluorine mass-balance protocols, expanded open HRMS and collision cross-section libraries, automated annotation workflows, improved quantification strategies for compounds lacking authentic standards, and hazard-informed approaches that better align monitoring and regulation with the chemical complexity and unidentified fraction of PFAS contamination.

## 1. Introduction

Per- and polyfluoroalkyl substances (PFAS) are a diverse group of synthetic fluorinated chemicals widely used since the 1950s in industrial processes and consumer products due to their exceptional stability, heat resistance, and surfactant properties [1,2]. Their molecular structure is defined by strong carbon–fluorine (C-F) bonds, making PFAS highly persistent in the environment and resistant to degradation [3,4]. Because of their widespread use and mobility, PFAS are now detected in water, soil, air, and biota, including in remote Arctic environments, as well as in human blood and placental tissues worldwide [5,6,7]. Human and environmental exposure involves complex mixtures of numerous PFAS, but current monitoring typically quantifies fewer than 100 compounds [8,9,10]. Moreover, analytical coverage remains biased toward well-characterized PFAS for which reference standards and established methods are available, whereas newer and less-studied variants can be more difficult to detect, leaving a substantial portion of the broader PFAS chemical space insufficiently characterized [11]. Between 2009 and 2017, a total of 455 newly identified PFAS were reported [12], consisting of approximately 45% anionic species, 17% cationic compounds, 29% zwitterions, and 8% neutral molecules [13]. While several well-studied PFAS such as perfluorooctanoic acid (PFOA), perfluorooctanesulfonic acid (PFOS), perfluorohexanesulfonic acid (PFHxS), and perfluorononanoic acid (PFNA) have been extensively characterized in terms of occurrence, toxicity, and treatability, the majority of PFAS in use today do not fall into this category [3,5,14].

“Unknown PFAS” in this review refers to substances for which analytical evidence, such as the signals in suspect and non-target HRMS-based screening or oxidative transformation of precursors, indicates per- or polyfluorinated structures but whose structures have not been confirmed, or for which no reference standard is available [15,16]. The amount of extractable organic fluorine (EOF) measured in environmental samples often exceeds the total targeted PFAS expressed as fluorine equivalents; this difference is referred to as “unidentified organofluorine” because TF also includes inorganic fluorine [17]. The difference between total fluorine and the target PFAS is reported as “unidentified fluorine”. However, these fractions are not equivalent to “unknown PFAS”. Unidentified organofluorine includes known but unmeasured PFAS, fluorinated pharmaceuticals and pesticides, inorganic fluorine compounds, and unknown PFAS [17,18]. The distinction between known and unknown PFAS highlights a significant scientific and regulatory gap [19].

Legacy PFAS such as PFOA and PFOS have established toxicological profiles, regulatory limits, and relatively well-characterized environmental pathways. In contrast, unknown PFAS are often overlooked during routine monitoring, since they cannot be quantified using conventional targeted methods, and may undergo complex transformation processes that yield additional unidentified intermediates [20,21]. The toxicological profile of unknown PFAS has not been characterized to a considerable extent. For this reason, the total risk originating from PFAS can be underestimated [13,14].

As regulatory agencies worldwide tighten standards for PFAS in drinking water and the environment, the presence of large quantities of unidentified PFAS poses major challenges [22]. Despite growing interest, comprehensive reviews addressing the occurrence, behavior, and detection of unknown PFAS or unidentified organofluorine remain scarce. Most existing reviews focus either on targeted occurrence trends or on advances in analytical instrumentation, without systematically linking unidentified organofluorine and unknown PFAS to treatment performance, regulatory compliance, and engineering decision making [23,24,25,26].

The novelty of this review lies in integrating these complementary lines of evidence across environmental and engineered systems and distinguishing structurally unidentified PFAS, unknown precursors and transformation products, and unidentified organofluorine fractions that cannot be fully explained by targeted analysis. To address this need, the present comprehensive review provides an evaluation of unknown PFAS, including: (i) pathways and sources contributing to the presence of unknown or emerging PFAS in environmental systems, (ii) analytical advancements and limitations in detecting and characterizing unknown PFAS, and (iii) key knowledge gaps and future research directions required to support effective monitoring and regulation.

## 2. Occurrence of Unknown PFAS and Unidentified Organofluorine in Water, Soil and Biota

Unknown PFAS and unidentified organofluorine have been detected across all major environmental compartments (Table S1), and in many studies, unidentified organofluorine represents the dominant fraction of total PFAS, demonstrating that the environmental and toxicological burden of PFAS is significantly underestimated when assessments rely solely on a limited set of legacy analytes.

**Figure 1:**
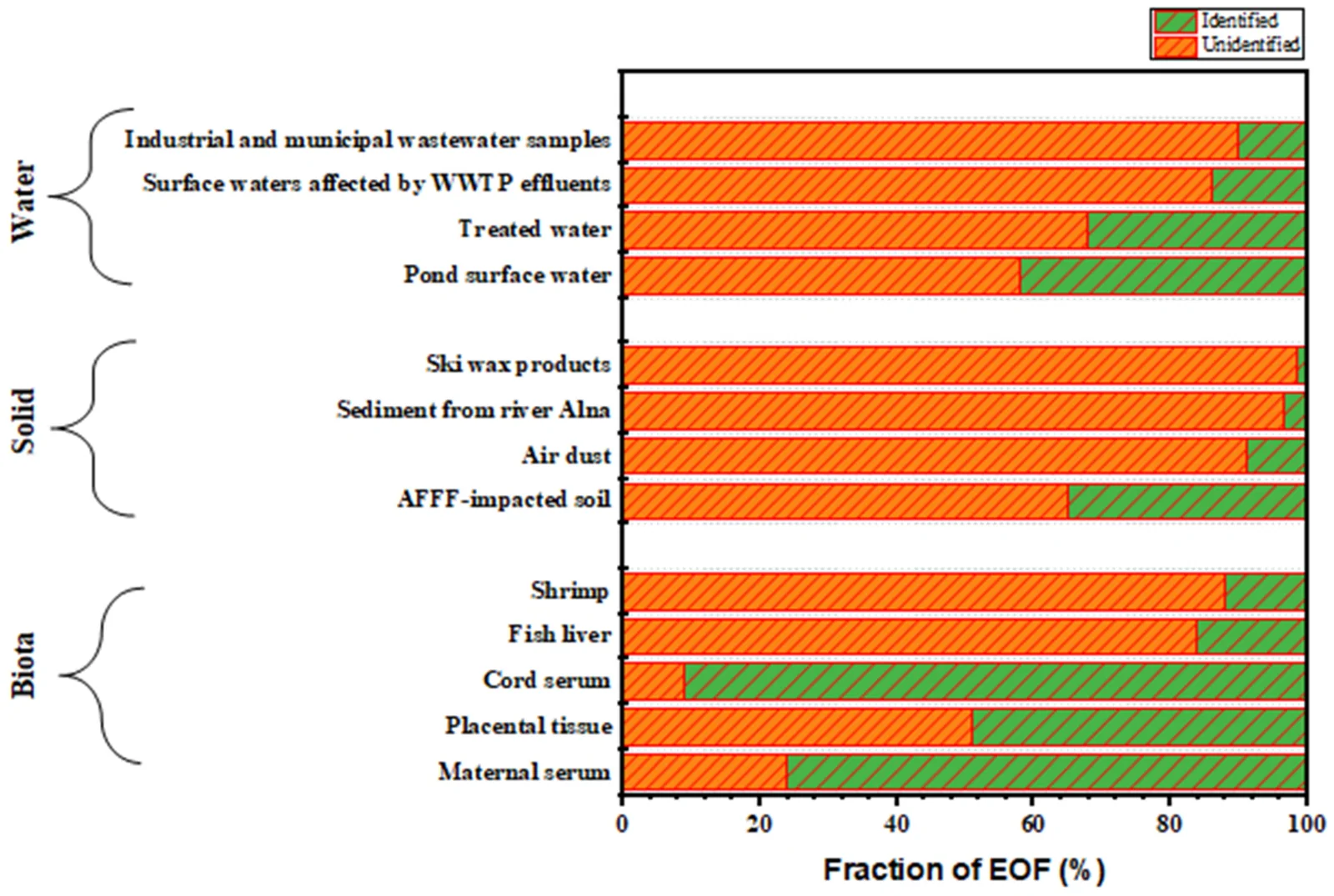
Percentage fractions of unidentified and targeted/identified PFAS in different environmental and biological matrices [28,29,30,31,32,33,34,35].

Figure 1 shows the percentages of unidentified EOF and targeted PFAS across different environmental media based on the EOF mass balance approach. The proportion of unidentified EOF ranges from 9% to 98.5%, depending on the matrix and the source of contamination. The figure shows that previous studies using EOF reported an unidentified EOF fraction generally higher than the defined one across different matrices. When the studied matrices are divided into solid, aqueous, and biota, the unidentified EOF ratio tends to be highest in solids. However, this generalization may not hold in every case because PFAS types, the number of target PFAS, extraction methods, and other analytical conditions vary. Also, EOF measures other organofluorine chemicals, such as fluorinated pharmaceuticals and pesticides, which can overestimate unknown PFAS [18,27]. The unidentified fraction is particularly prevalent in matrices affected by environmental and anthropogenic sources. This indicates that current PFAS monitoring methods can detect only a small portion of the total organofluorine load and that non-targeted screening approaches are important for comprehensive risk assessment.

Analytical methods that measure total fluorine (TF) using combustion ion chromatography (CIC) and extractable organofluorine (EOF) via solid-phase extraction (SPE) followed by CIC offer a mass balance perspective on organofluorine contamination. For EOF-based mass balance, targeted PFAS concentrations are converted to fluorine-equivalent concentrations (${\sum F}_{target})$ and compared with measured EOF; the ratio ${\sum F}_{target}/\mathrm{EOF}\;\times \;100$ represents the percentage of extractable organofluorine explained by targeted PFAS, whereas the difference (EOF − ${\sum F}_{target}$) represents the unidentified extractable organofluorine [36].

### 2.1. Water Matrices

Water matrices including drinking water, surface water, groundwater, wastewater, landfill leachate and water from aqueous film-forming foam (AFFF)-impacted sites are among the best studied compartments for unknown PFAS due to the relatively straightforward sample preparation and the availability of sensitive analytical tools. In many drinking water and surface water samples, targeted PFAS accounted for approximately 40% of the measured organic fluorine, leaving ~60% unidentified and indicating a substantial unidentified organofluorine fraction that may include unknown PFAS and other fluorinated compounds [36]. Idowu et al. [32] reached similar conclusions in a systematic review of total PFAS methods, found TF, EOF, and TOPA to be reported in 38%, 34%, and 31% of studies, respectively, and concluded that a substantial fraction of measured fluorine frequently remains unaccounted for, highlighting the value of combining fluorine mass-balance approaches with non-target screening to investigate unknown PFAS and other organofluorine species in water matrices.

#### 2.1.1. Drinking Water and Raw Water

PFAS are commonly detected in aquatic systems, but conventional analytical methods capture only a limited range of PFAS, and many unknown PFAS remain undetected [37]. Pilot EOF studies in drinking water have revealed that unknown PFAS can dominate the organofluorine mass [29]. For example, Jiao et al. [29] reported that in samples from drinking water treatment plants around Taihu Lake, China, at least 68% of EOF remained unidentified and unexplained by targeted PFAS, even after including 34 legacy and emerging PFAS compounds in the targeted analysis. This discrepancy arises because targeted methods quantify only predefined PFAS for which suitable analytical standards and methods are available, while unidentified precursors, transformation products, and other fluorinated compounds remain outside the analytical target list [29]. He et al. [38] improved EOF recovery (93%) for highly mobile PFAS such as trifluoroacetic acid (TFA) yet still observed sizeable fractions of unidentified EOF (67%) in treated drinking waters, implying that many small, mobile PFAS and their transformation products are not captured by current target lists. These findings are consistent with recent national-scale assessments (e.g., high TFA and other ultrashort-chain PFAS in tap water) and suggest that rarely monitored, highly mobile PFAS may be especially relevant for drinking water exposure [39].

Raw water used as a drinking-water source may also receive complex PFAS mixtures from upstream industrial discharges and other contaminated source areas. For example, recent studies illustrate the magnitude of this unidentified burden: in surface waters from Norway, 84–99% of EOF was of unidentified origin, while a recent US drinking water study found that 37 targeted PFAS accounted for only 6–68% of EOF across sampled systems [40,41]. Recent long-term PFAS-specific non-target analysis (NTA) monitoring further revealed that even continuous monthly sampling can uncover large numbers of previously unknown PFAS, as demonstrated by Ulrich et al. [42], who identified 55 PFAS, many of which were first reported in water downstream of a fluoropolymer producer, underscoring the likelihood that similar unknown PFAS also enter raw drinking water sources. Similarly, Wang et al. [43] applied target, suspect, and non-target screening throughout a drinking-water system and detected 17 PFAS homologous series comprising 51 homologues by non-target screening and 50 tentatively identified PFAS by suspect screening. Fifteen PFAS were assigned probable structures with relatively high confidence, including seven reported for the first time in a drinking-water system, while semi-quantified non-target PFAS ranged from 4.10 to 17.6 ng/L. Notably, both target and non-target PFAS persisted into household tap water with negligible removal during conventional treatment. These findings demonstrate that drinking-water sources and finished waters contain a substantial and chemically diverse PFAS fraction that remains unidentified by conventional targeted monitoring, underscoring the need to integrate fluorine mass-balance approaches with suspect and non-target screening for more comprehensive exposure assessment.

#### 2.1.2. Surface Waters and Groundwater

PFAS can enter surface water and groundwater from many sources, such as industrial activities, wastewater discharge, urban runoff, and the use of AFFF, and can be transported to other environmental media. A global assessment compiling more than 45,000 surface-water and groundwater samples showed widespread PFAS contamination and further indicated that current monitoring likely underestimates the environmental PFAS burden because only a limited suite of compounds is routinely quantified [44]. Marine currents and sea-spray aerosols can carry PFAS across long distances because these compounds accumulate in surface microlayers and exhibit strong surfactant behavior [45,46]. Fluorine mass balance studies in rivers and reservoirs impacted by wastewater effluent, industrial discharge, or urban runoff indicate that known PFAS often represent only a small fraction of the organofluorine. For example, Nxumalo et al. [31] combined TF, EOF, and targeted PFAS analysis in surface waters and showed that unidentified organofluorine frequently dominated (>90%) the fluorine mass, particularly downstream of wastewater treatment plants and industrial sources. Non-targeted PFAS screening using LC-HRMS, including fragment ion flagging and mass defect filtering, has identified numerous previously unreported PFAS classes (e.g., ultrashort-chain acids, novel fluorotelomer derivatives, ether/iodinated PFAS), but the large number of unidentified features indicates that a substantial portion of PFAS in surface waters is still unknown [47]. Similarly, Miyake et al. [17] found that around 60–90% of TOF in marine water remains unidentified. A wide range of novel PFAS have already been identified in surface waters by suitable PFAS-specific NTA approaches [48,49,50]. Similarly, Liwara et al. [51] combined expanded target analysis with suspect and de novo non-target screening (SNTS) using liquid chromatography-tandem mass spectrometry (LCMS/MS) and time-of-flight mass spectrometry (TOF-MS) to investigate Dutch surface waters and demonstrated that conventional monitoring substantially underestimates PFAS diversity. By incorporating newly available sulfonamide standards and collision cross-section (CCS) data, the study identified 19 confidently annotated compounds through SNTS, including fluorinated pharmaceuticals, pesticides, an unsaturated PFOS analogue, and 6:2 FtSO_2_AoS confirmed at level 1a. These findings illustrate how integration of ion mobility and advanced screening workflows reveals unexpected and previously unreported PFAS and other organofluorine compounds in European surface waters that fall outside routine monitoring programs. In another study, Joerss et al. [49] identified 86 PFAS including numerous emerging and novel PFAS in German and Chinese rivers affected by industrial point sources, revealing country-specific contamination signatures and demonstrating that many unregulated PFAS are emitted continuously into surface water systems. Kirkwood-Donelson et al. [52] identified the candidate structures of 11 unknown PFAS, including various per- and polyfluorinated ether sulfonic acids and multiheaded perfluorinated ether acids, from an aquatic passive sampler deployed in the Cape Fear River downstream of a fluorochemical plant using liquid chromatography-ion mobility spectrometry collision induced dissociation mass spectrometry (LC-IMS-CID-MS). Groundwater, especially near AFFF-impacted sites, airports, military bases, and fluorochemical production facilities, often exhibits complex PFAS fingerprints dominated by precursors and transformation products that are not part of routine monitoring suites. Non-target Liquid Chromatography-Quadrupole Time of Flight Mass Spectrometry (LC-QTOF) analysis of historical AFFF formulations and AFFF-impacted groundwater revealed 40 previously unreported PFAS classes and detected 17 previously reported classes for the first time, demonstrating that a substantial portion of PFAS mass at contaminated sites remains unidentified. Of the 40 newly identified classes, 34 were attributed to electrochemical fluorination (ECF), an industrial fluorination process that produces a complex mixture of linear, branched and structurally related perfluorinated compounds [53]. D’Agostino et al. [54] employed weak anion (Oasis WAX) and weak cation (Oasis WCX) exchange cartridges to fractionate organofluorine species in AFFF and surfactant formulations based on ionic properties. This strategy separated acidic, neutral, and cationic constituents, enabling functional group assignment and subsequent qualitative analysis. The approach led to the identification of 22 classes of previously unidentified fluorinated compounds, including novel structures containing tertiary and quaternary ammonium groups combined with carboxylate, ether, and amide functionalities. Reviews of PFAS in subsurface environments highlight the prevalence of polyfluorinated species and polymer-derived PFAS that are difficult to identify and quantify, underscoring the importance of non-target analysis to detect unknown PFAS in aquifers [55]. Similarly, non-targeted assessments in Taihu Lake revealed numerous emerging PFAS including bis (trifluoromethanesulfonyl)imide (Ntf_2_) and the fluorinated ether acid C_7_HFPO-TA whose spatial distribution and increasing temporal trends indicate active transport from industrial sources to downstream surface waters [56].

#### 2.1.3. Wastewater, Industrial Effluents and Landfill Leachate

Municipal and industrial wastewater are key point sources of both known and unknown PFAS to aquatic environments. In wastewater treatment plant (WWTP) effluent and raw wastewater samples, unidentified organofluorine accounted for 48–73% and 9–89% of AOF, respectively [57]. Wang et al. [58] applied suspect screening and homologue analysis using quadrupole TOF MS to influent and effluent from a WWTP at a fluorochemical facility near the Yangtze River and identified 90 PFAS across 15 classes, including 37 previously unreported PFAS. Ruyle et al. [59] further demonstrated that conventional PFAAs represented only a minor fraction of the broader organofluorine burden in eight large U.S. WWTPs: 18 measured PFAAs accounted for 11 ± 8% and 21 ± 12% of EOF in influent and effluent, respectively, while PFAA precursors estimated by TOP coupled with Bayesian inference accounted for 13 ± 5% and 16 ± 13%, respectively. More importantly, targeted analysis explained less than 25% of the total PFAA precursor burden estimated using TOP coupled with Bayesian inference, while suspect screening of >1000 additional precursors identified only six additional structures, indicating that the molecular identities of most PFAA precursors in municipal wastewater remain unidentified [59].

In electronics fabrication wastewater, nontarget HRMS detected 41 homologous PFAS series comprising 133 individual homologues, revealing a large suite of previously unmonitored PFAS. Six of these series were newly reported for the first time, and semi-quantification showed that nontarget PFAS dominated total PFAS mass, with combined target and nontarget concentrations reaching up to 78,700 ng/L [60]. Semiconductor manufacturing has recently emerged as a major source of previously unrecognized PFAS. Chen et al. [61] applied a fragment-based nontarget workflow to wastewater from five semiconductor facilities and identified 20 PFAS across 12 subclasses, 15 of which had never been reported in semiconductor sewage before. Dominant compounds were perfluorobutane-sulfonamido derivatives (e.g., FBSE, FBSA, FBSEE-diol) with concentrations of up to 482 μg/L, indicating the presence of large quantities of replacement PFAS in industrial wastewater. Their TOP-based precursor analysis also showed aerobic transformation of fluorinated alcohols to fluorinated acids, suggesting that semiconductor wastewaters contain significant pools of uncharacterized precursors that convert into ultrashort-chain PFAAs during treatment.

Non-target analysis has revealed previously unreported PFAS in various industrial wastewaters. For instance, 22 newly identified PFAS were found in fluorochemical industry wastewater and three in textile wastewater [62,63]. Further details on the newly identified compounds, including their names, confidence levels, identification methods, and contribution to total PFAS, are given in Table S2. A large-scale survey in China further showed that wastewater from semiconductor facilities is a major source of emerging PFAS and unknown precursors. Qiao et al. [64] identified 82 PFAS including 13 PFAS classes reported for the first time globally in semiconductor wastewater, with total PFAS levels reaching 12 μg/L, far higher than municipal wastewater (25–950 ng/L). Their TOP assay results also revealed 0.043–0.83 nmol/L unidentified precursor pools that cannot be captured by target methods.

The newly reported PFAS in industrial samples (Table S2) were identified at varying confidence levels, mostly levels 2–3. Compounds at these levels were not confirmed with authentic reference standards (except level 1 identification); therefore, these identifications are tentative. In addition, concentrations of compounds with no reference standard can be estimated semi-quantitatively using a structurally similar standard [62]; however, because of differences in compound ionization efficiency, matrix effects, and measurement methods, these estimates are uncertain, and large errors have been reported [65]. A recent NTA program at the Chemours Fayetteville Works (NC) further demonstrated the extreme complexity of wastewater derived from PFAS, identifying more than 250 previously unknown PFAS features in 21 general wastewater facilities [66]. Of these, 255 unique unknown PFAS were observed across process wastewater and general facility discharges, with only 12 species overlapping between the two streams, indicating highly source-specific PFAS signatures. Recently, Larsson et al. [67] applied a multisorbent solid-phase extraction approach combined with LC-HRMS and fluorine mass balance analysis to municipal wastewater and demonstrated that unidentified organofluorine extends beyond highly fluorinated PFAS. While 23 highly fluorinated PFAS and precursors were detected at low to sub-ppt levels, 30 low-fluorinated pharmaceuticals and transformation products were identified at concentrations up to three orders of magnitude higher. Despite containing <30% fluorine by mass, these compounds contributed 28–42% of the EOF, with sitagliptin, bicalutamide, and celecoxib carboxylic acid as major contributors. In addition, coextracted inorganic fluoroanions such as hexafluorophosphate (PF_6_^−^) and tetrafluoroborate (BF_4_^−^) accounted for 7–19% of EOF, demonstrating that part of the apparent unidentified EOF can arise from inorganic fluorinated anions rather than unknown PFAS. Collectively, these findings support the growing recognition that municipal and industrial wastewaters contain complex mixtures of identifiable and unidentified terminal PFAS, precursor compounds, and partially transformed intermediates that remain inadequately characterized by conventional targeted monitoring [68,69].

Landfill leachate and industrial effluents typically contain complex mixtures of legacy PFAS, polymeric PFAS, and numerous transformation products. Fluorine mass balance studies in these matrices consistently show large gaps (75–99%) between EOF and known PFAS, confirming that waste management systems are major sources of unidentified organofluorine to downstream waters [70,71]. Long-term NTA studies in fluorochemical industrial zones support this interpretation; for example, Fu et al. [56] identified 58 emerging PFAS in wastewater and surface waters around Taihu Lake, while a companion study detected 83 PFAS, 22 reported for the first time in effluents and receiving waters, demonstrating that industrial discharges continuously introduce novel PFAS such as C7 HFPO-TA at exceptionally high levels (447,000 ng/L). Overall, current evidence indicates that in most water matrices the majority of organofluorine often resides in unidentified PFAS or other fluorinated organics, highlighting the need for continued development of non-targeted analytical approaches and group-based regulatory concepts [32].

Atmospheric emissions further complicate the role of WWTPs and landfills as PFAS sources. Wang et al. [72] quantified both neutral and ionizable PFAS in air samples collected from a municipal WWTP and landfill and reported that neutral PFAS (Σn-PFAS: 2816–564,343 pg/m^3^) exceeded ionizable PFAS concentrations. Dominant species included 8:2 fluorotelomer alcohol (FTOH) among neutrals and TFA among ionizable compounds. Importantly, total oxidizable precursor (TOP) analysis revealed that unidentified precursors accounted for 46.4–58.4 mol % of total PFAS in the air samples over landfill and WWTP [72], demonstrating that substantial unknown PFAS fractions are emitted via atmospheric pathways from wastewater and landfill systems.

### 2.2. Solid Matrices

Solid matrices such as soils, sediments, biosolids, sewage sludge, and landfill solids act both as sinks and long-term secondary sources of PFAS, including unidentified compounds and precursor species [73,74].

#### 2.2.1. Soil and Sediment

Primary inputs include land application of biosolids, irrigation with contaminated water, AFFF release, atmospheric deposition of volatile precursors, and accumulation of PFAS-containing consumer products in landfills [55]. Early fluorine mass-balance studies demonstrated the magnitude of this unidentified organofluorine accounting for >56% and >95% of EOF in surface sediments and soils, respectively, leaving a substantial unidentified fraction potentially comprising unknown PFAS, precursors, and other fluorinated compounds [75,76]. Studies across urban, agricultural, and remote regions consistently detect PFAS in soil and sediments, with concentrations often correlated with population density and land-use intensity. Lyu et al. [55] reported widespread PFAS occurrence in subsurface environments and noted that many chromatographic features consistent with PFAS remained unidentified due to the lack of reference standards and structural information. More direct evidence of hidden precursor burdens has recently emerged from contaminated soils. Using an optimized direct total oxidizable precursor (dTOP) assay at a site near the fluorochemical production site in Flanders, Belgium, Salangad et al. [77] estimated 192 nmol/kg of unidentified precursors at Blokkersdijk, representing 72% of the post-oxidation PFAS burden, and 210 nmol/kg at Vlietbos, representing 82%, whereas targeted analysis detected only 0.6 nmol/kg of known precursors (FOSA and Et-FOSAA) at the latter site. These discrepancies indicated that oxidizable precursor pools in highly contaminated soils can be dominated by non-target and/or structurally unknown PFAS.

#### 2.2.2. Biosolids

Biosolids represent important reservoirs of analytically unidentified organofluorine because conventional targeted methods capture only a portion of the fluorinated chemical burden. A United States Geological Survey (USGS) study in New Hampshire showed that biosolids and associated soils contained a wide range of PFAS and that their mobility and solid–water partitioning were influenced by both compound properties and soil characteristics; however, comprehensive organofluorine mass balances were not achieved, suggesting that additional, unknown PFAS classes or other organochlorine compounds may be present [78]. Spaan et al. [27] reported that 73% of the EOF was unidentified in WWTP sludge and that it may contain pharmaceuticals, pesticides, fluorinated polymers, and neutral PFAS. This large analytical gap is consistent with Fredriksson et al. [79], who found that AOF concentrations in biosolids exceeded targeted PFAS by factors of 3–20, particularly in anaerobically digested solids, where ≥90% of fluorine remained uncharacterized. Non-target screening reinforces this pattern: Muñoz et al. [80] detected over 80 suspect PFAS including ether-PFAS, fluorotelomer surfactants, and sulfonamide derivatives, many of which lacked database matches, indicating structurally novel or polymer-derived unknowns. Dickman et al. [81] similarly observed that targeted PFAS represented only 20–35% of AOF, with numerous persistent, thermally stable unknown PFAS surviving drying and pelletization processes. Schaefer et al. [82] showed that biosolid-amended soils release substantial amounts of PFAAs, with more than 75% of the PFAS fluorine in the biosolid and precursor transformations generating additional PFAA over time. Collectively, these findings indicate that biosolids act as reservoirs and long-term sources of unidentified organofluorines that may contain unknown PFAS to terrestrial and aquatic environments.

#### 2.2.3. Landfills and Solid Waste

Landfills receive large quantities of PFAS-containing consumer products (e.g., stain-guarded textiles, paper and food packaging, carpets, firefighting foams, fluoropolymer-coated items), making them long-term secondary sources of both known and unknown PFAS. Detailed mass balance work shows a strong mismatch between what enters landfills (dominated by precursor-rich chemistries such as diPAPs and FTOHs in paper, textiles and carpets) and what is measured in leachate (dominated by perfluoroalkyl acids and fluorotelomer acids). This shift suggests extensive transformation within the waste matrix, while semi-volatile, polymeric, and other poorly characterized PFAS may remain inadequately captured by conventional target analysis [83]. Recent analysis of landfill organics across multiple depths and locations found 29 PFAS from six classes (PFCAs, PFSAs, FTCAs, PASFs, FTSs, PFECAs) at 6.7–73 µg/kg, with PFCAs and FTCAs accounting for up to 82% and 40% of total PFAS, respectively, and clear vertical “hot zones” at intermediate depths where PFAS concentrations, total carbon and dissolved organic matter co-peaked [84]. Emerging work directly on landfill refuse further indicates how much PFAS mass remains present in solids as unidentified precursors. In a closed municipal solid waste landfill, Yu et al. [85] quantified 24 target PFAS (18 PFAAs and 6 precursors) in aged refuse and applied a direct TOP assay to estimate the additional burden from oxidizable precursors. Unknown precursors increased apparent PFAS contamination by a factor of 4–18, with long-chain PFCAs and precursor species largely retained in refuse even after more than a decade of leaching. This implies that standard targeted monitoring of leachate alone can severely underestimate the total PFAS stock and future emission potential of landfills, particularly for unknown precursor pools that reside in partially degraded waste and organics. Leachate measurements provide a complementary view of how these precursor reservoirs evolve during waste degradation. In a survey of 17 landfills in Washington State, Capozzi et al. [86] measured 32 PFAS before and after TOP assay and found 5:3 FTCA to be the dominant PFAS in leachate, consistent with degradation of diPAPs and other precursors in carpets, textiles and food packaging. Total PFAS ranged from tens to ~1.7 × 10^5^ ng/L, but TOP results suggested that only small amounts of unknown oxidizable precursor remained in the aqueous phase and that chain-shortening reactions during oxidation could even reduce overall PFAS mass [86]. Together with broader syntheses showing that diPAPs/FTOHs entering with waste and FTCAs/PFCAs leaving in leachate are systematically different [83], this indicates that precursor transformation, solid-phase retention, and partitioning collectively reshape the PFAS pool within landfills, leaving leachate comparatively enriched in mobile transformation products. Levalier et al. [87] provided excellent evidence for matrix effects in landfill leachate, although they investigated EOF/SPE rather than AOF.

Beyond leachate and solids, landfill gas has recently been recognized as a key pathway for neutral and semi-volatile PFAS including many species that are effectively unknown from a water-monitoring perspective. Saha et al. [84] showed that PFAS associated with landfill organics covary with carbon content and moisture, supporting the idea that PFAS-laden organic matter can act as a source for both aqueous and gaseous releases as degradation proceeds [84]. Building on earlier site-specific work, more recently, a nationwide campaign by De la Cruz et al. [88] characterized volatile PFAS in landfill gas from 30 U.S. landfills across diverse climates and used these data to estimate annual PFAS emissions via landfill gas, demonstrating that volatile PFAS releases are a non-negligible component of the national PFAS budget.

### 2.3. Biota

Biota integrate PFAS exposure over time through bioaccumulation and are thus critical sentinels for both known and unknown PFAS. Because many PFAS bioaccumulate or biomagnify, especially in apex predators, organofluorine measurements in tissues often reveal large fractions of unidentified EOF, indicating the potential presence of unknown bioaccumulative PFAS [89,90]. Nontargeted LC–ion mobility spectrometry–mass spectrometry (LC-IMS-MS) coupled with the PFAS IMplementer for Mass Spectrometry (PIMMS) revealed a substantial burden of previously unknown PFAS-related features in human serum. Among 15 human serum samples, PIMMS prioritized 141 validated halogenated features, including 10 likely library-matched compounds, 17 tentative suspect matches, and 141 initially unmatched features after filtering; structural elucidation ultimately identified two novel PFAS that would not have been captured by conventional targeted analysis [91]. These included an iodinated PFAS with the molecular formula C_5_H_5_F_3_O_2_ and a brominated PFAS with the formula C_5_H_7_F_3_BrO_2_, demonstrating the occurrence of unconventional mixed-halogen PFAS in human serum. Human blood samples and shrimp samples had 15 to 70% and approximately 89% of unidentified organofluorine, respectively, in EOF [92,93]. Spaan et al. [89] performed fluorine mass balance and suspect screening on marine mammals from the Northern Hemisphere and found that targeted PFAS explained only part of the EOF in liver tissues; suspect screening revealed dozens of additional PFAS structures across multiple classes, yet a substantial fraction of EOF remained unidentified. More recently, Lauria et al. [90] substantially reduced the fluorine mass balance gap in marine mammals by combining HRMS and NTA with machine learning-based ionization efficiency (IE) prediction. In their study of liver tissues from pilot whales (East Greenland) and white-beaked dolphins (West Greenland and Sweden), up to 70% of EOF could be unknown PFAS. By applying machine-learning IE prediction to quantify 35 suspect PFAS (confidence level 2–4) lacking analytical standards, the fraction of unidentified EOF was reduced from up to 70% to 0–27%, demonstrating that many previously unquantified PFAS can be semi-quantified when advanced computational tools are incorporated into PFAS workflows [90]. These findings challenge the traditional view that PFAS exposure is dominated by water-soluble anionic species and indicate that unknown PFAS with different partitioning behavior may make a substantial contribution to overall body burdens. Avian species, particularly top predators, are also key indicators of unknown PFAS. A comprehensive temporal study of white-tailed sea eagle (Haliaeetus albicilla) eggs from Sweden (1969–2021) demonstrated that although targeted PFAS accounted for the majority of extractable organofluorine (EOF; 81–100%), high-resolution suspect screening still identified 55 additional unknown PFAS, 43 of which were not included in standard target lists [94]. Among these, a homologous series described by the formula [C_n_F_2n-9_H_9_NO_4_S]^−^ (n = 12–16) showed clear PFAS-like fragmentation but could not be structurally assigned and was therefore classified with Confidence Level 5, indicating that only its exact molecular features and diagnostic mass-spectrometric evidence were available, while its chemical structure remained unidentified; thus, it may represent unknown PFAS. These findings illustrate that even when EOF suggests near complete mass balance, low abundance but structurally novel PFAS remain undetected by routine monitoring. Fluorine mass balance approaches have also been applied to human blood and serum to assess exposures to total organofluorine. Cioni et al. [95] compared TF, EOF, and targeted PFAS in human serum and observed that targeted PFAS accounted for a large but incomplete fraction of EOF, indicating that humans are exposed to both measured and unidentified organofluorine. Similarly, Yeung and Mabury (2016) [96] quantified 52 PFAS in human plasma and whole blood from Germany and China and compared them to EOF, showing that targeted PFAS explained only 31–86% of EOF in Chinese samples and 52–100% in German samples. Notably, an increasing proportion of unidentified organofluorine was observed in samples collected after 2000, suggesting growing human exposure to newly developed or previously unrecognized fluorinated compounds.

Collectively, the magnitude and composition of the unidentified organofluorine fraction are strongly matrix and source dependent rather than being uniform across different environmental compartments. Industrial wastewater and AFFF-impacted waters generally contain diverse source-specific precursors and replacement chemistries, whereas drinking and surface waters increasingly reflect dilution, transformation, treatment, and preferential transport of mobile PFAS. In soils, sediments, biosolids, and landfill solids, solid-phase retention favors hydrophobic, precursor, and polymer-associated fluorinated species that may be poorly represented by conventional extraction and targeted analysis. Biota further modify this chemical profile through selective bioaccumulation and biotransformation. Consequently, differences in unresolved fluorine among matrices reflect not only analytical coverage, but also source chemistry, environmental transformation, partitioning, mobility, and treatment history.

## 3. Analytical Approaches for Unidentified Organofluorine and Unknown PFAS

**Figure 2:**
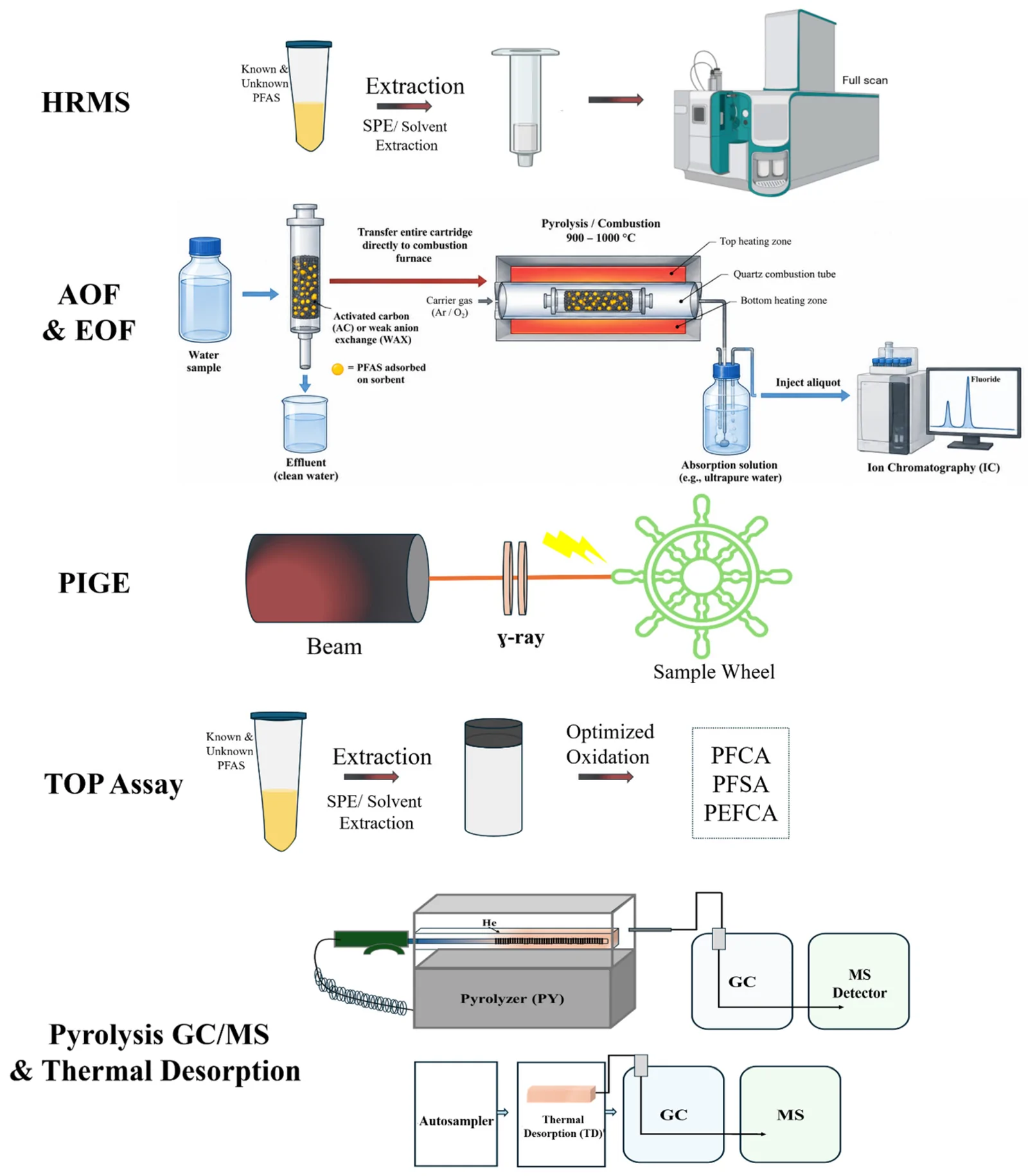
Analytical approaches for the detection of unknown PFAS in environmental samples. “Analytical approaches for the detection of unknown PFAS in environmental samples” created in BioRender. Ahmed, F. (https://BioRender.com/0afwp3a, accessed on 14 October 2026) is licensed under CC BY 4.0.

Detection of unknown PFAS requires an integrated analytical strategy because no single method captures the full diversity of ionic, neutral, zwitterionic, ultrashort-chain, precursor, and polymeric PFAS. Sample preparation is therefore a critical first step because extraction and cleanup determine which compounds are recovered, enriched, or lost before instrumental analysis. Matrix constituents such as proteins, lipids, humic substances, and salts can further influence extraction efficiency and ionization, thereby affecting HRMS feature detection, fluorine mass-balance interpretation (EOF/AOF/TF), and precursor oxidation assays [97]. Consequently, pretreatment bias can substantially alter the apparent composition of the unknown PFAS fraction. Current approaches include liquid–liquid extraction (LLE), solid-phase extraction (SPE), weak-anion-exchange SPE (WAX-SPE), accelerated solvent extraction (ASE), supercritical fluid extraction (SFE), digestion procedures, and sorbent-assisted adsorption-pyrolysis methods (Table S3). Selection of the pretreatment method should therefore be matrix-specific and matched to the analytical objective, whether targeted quantification, suspect/non-target screening, fluorine mass balance, or precursor assessment. The principal analytical approaches used to characterize unknown PFAS are summarized in Figure 2.

**Figure 3:**
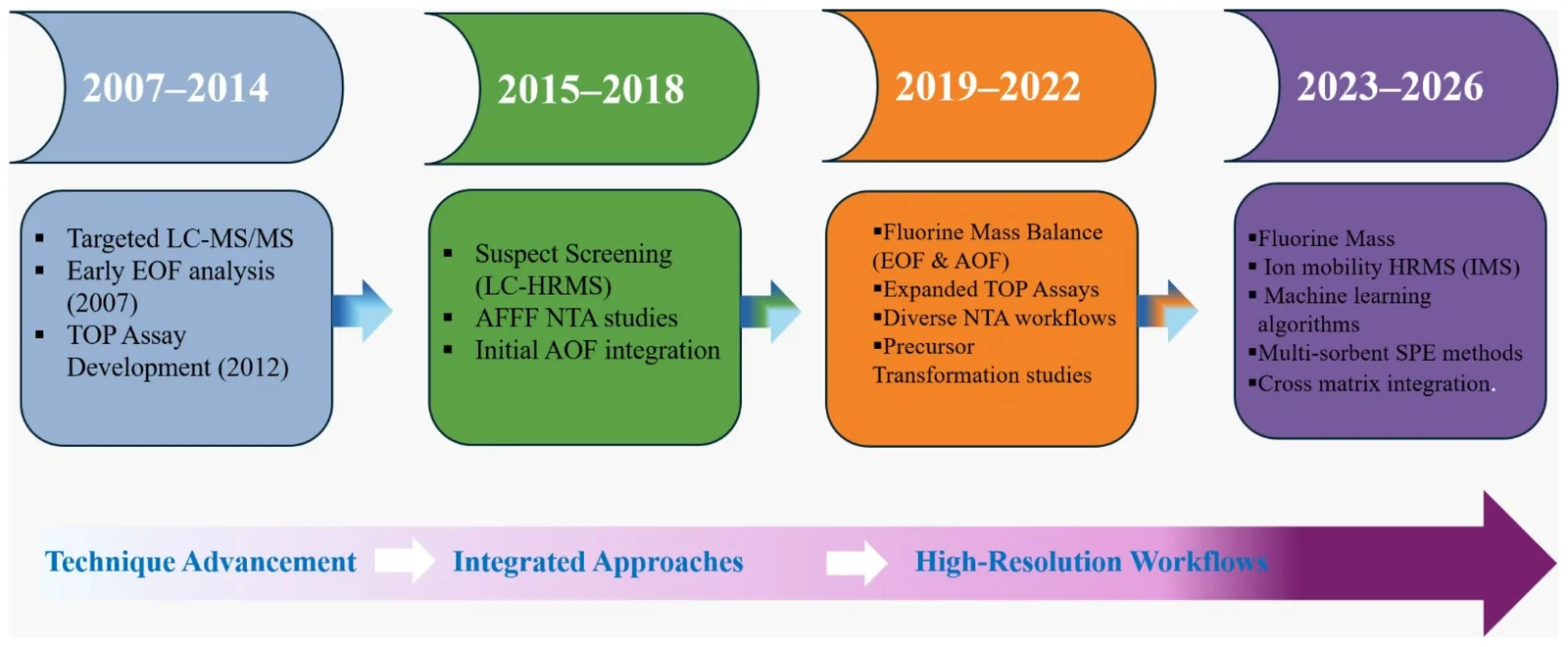
Evolution of analytical strategies for detecting unknown PFAS in environmental matrices (2007–2026) [16,53,91,98,99,100,101].

Analytical strategies for unknown PFAS have evolved substantially over the past two decades (Figure 3). Early efforts (2007–2014) relied primarily on targeted LC-MS/MS, extractable organofluorine (EOF) measurements, and development of the total oxidizable precursor (TOP) assay. During 2015–2018, suspect screening and non-target analysis (NTA) using high-resolution mass spectrometry (HRMS) expanded the detectable PFAS chemical space, while subsequent advances (2019–2022) increasingly integrated fluorine mass-balance approaches (EOF/AOF), expanded TOP assays, and diverse NTA workflows. More recent strategies (2023–2026) emphasize multidimensional and integrated analysis, including ion mobility HRMS, machine-learning-assisted annotation, multisorbent extraction, and cross-matrix integration. Collectively, this evolution reflects a transition from measuring predefined PFAS toward complementary workflows designed to progressively constrain, prioritize, and structurally characterize the unidentified PFAS fraction [16,53,91,98,99,100,101].

### 3.1. High Resolution Mass Spectrometry (HRMS)

HRMS has become the cornerstone of modern PFAS discovery and is one of the most powerful tools currently available for identifying previously unrecognized fluorinated compounds in environmental matrices [102,103]. Unknown PFAS pose extraordinary challenges because they lack analytical standards, reference fragmentation libraries, and validated retention-time windows, and because their physicochemical diversity spans neutral, ionic, zwitterionic, polymeric, and highly oxygenated chemical classes. HRMS is not simply complementary to targeted quantification methods; it is the primary viable starting point for constructing the chemical inventory of unknown PFAS at environmentally relevant concentrations [36,47,53]. HRMS-based PFAS detection offers several advantages, including ultra-high resolution, high sensitivity, no reference-standard requirement, versatile ionization, and the ability to perform retrospective analysis on collected data [47,101,104].

#### 3.1.1. Rationale and Analytical Mechanisms

The rationale for HRMS-based PFAS detection lies in the exceptional stability and distinct elemental composition of fluorinated substances. Because PFAS contain multiple CF_2_ and CF_3_ units, they exhibit systematically predictable mass defects relative to hydrocarbon analogues. This enables their detection by mass defect filtering, Kendrick mass defect analysis (KMD), fluorine-number plotting, isotopic pattern analysis, Kaufmann analysis and homologous series clustering [105,106,107]. These analytical tools allow scientists to differentiate PFAS from other halogenated or oxygenated organic compounds even in the absence of standards. For example, the use of KMD-CF_2_ transformations facilitates the automatic grouping of PFAS homologous series, enhancing the discovery of ether-PFAS, ultrashort-chain acids, and fluorotelomer derivatives in environmental datasets [53,108]. Furthermore, exact-mass precursor ion selection followed by data-dependent or data-independent MS/MS fragmentation provides detailed structural information because PFAS show diagnostic neutral losses such as C_2_F_4_, HF, SO_3_^−^, COF_2_, and CF_3_^−^ [107]. These fragmentation pathways are instrumental in identifying functional groups such as sulfonates, carboxylates, amides, and ether linkages. As such, HRMS enables the reconstruction of core structural motifs even in the absence of authentic reference standards. Two primary HRMS workflows, namely suspect screening (SS) and NTA, are used to interrogate unknown PFAS. Suspect screening begins with a curated list of potential PFAS structures sourced from manufacturing inventories, production patents, chemical registries, import/export disclosures, and environmental release data [101,109]. These suspect lists may contain thousands of entries, including documented industrial PFAS, patented fluorinated surfactants, precursor formulations, and transformation-product predictions. SS increases analytical confidence because it compares detected masses to known chemical formulas or exact-mass predictions; however, it cannot detect compounds outside the predefined suspect list. As PFAS manufacturers frequently produce proprietary mixtures with undisclosed compositions, SS alone captures only a limited share of unknown PFAS. NTA is particularly powerful for identifying unknown PFAS because it does not rely on preconceived structural expectations. Instead, it uses machine learning-based fragmentation prediction, mass defect clustering, RT-PFAS modeling, and in silico libraries to generate possible structures [110,111]. Recent NTA workflows combining machine learning with HRMS have further expanded the structural landscape of PFAS by identifying ether-linked, highly oxygenated, and functionalized PFAS intermediates formed during biological or oxidative transformations. Barzen-Hanson et al. [53] identified more than 40 novel PFAS features in AFFF-impacted sites, many of which did not match any known PFAS standards or suspect-list entries.

#### 3.1.2. Structural Elucidation

Isomeric PFAS, such as branched and linear variants, show similar mass spectra but may differ markedly in toxicokinetics, sorption behavior, and membrane rejection performance, making their structural differentiation analytically important [112]. Tandem mass spectrometry (MS/MS) provides an additional level of structural information by isolating precursor ions and comparing product-ion spectra, diagnostic fragment-ion abundances, and characteristic neutral losses [113,114]. However, positional and branched/linear PFAS isomers can generate highly similar MS/MS spectra because they contain the same elemental composition and functional groups, and fragmentation may therefore be insufficient for unequivocal isomer assignment [114,115]. Differences in relative product-ion intensities, fragmentation pathways, and collision-energy-dependent fragmentation can improve discrimination for some PFAS isomers, particularly when authentic standards are available for comparison. Consequently, confident isomer identification generally benefits from combining MS/MS with chromatographic retention behavior and orthogonal information such as ion-mobility-derived collision cross section (CCS), rather than relying on accurate mass alone [116]. Additionally, many PFAS exhibit poor ionization efficiency, including zwitterionic PFAS, fluorinated amines, quaternary ammonium PFAS, and neutral PFAS precursors [117]. Consequently, polarity-switching acquisition (±ESI) and/or alternative ionization modes can be necessary to reduce “ionization bias” in discovery-oriented datasets [98,118]. Ultrashort-chain PFAS (e.g., TFA, PFPrA) elute near the chromatographic void volume and require specialized chromatographic conditions for retention; consequently, they are frequently missed in standard HRMS workflows [119].

Polymeric and oligomeric PFAS present a distinct challenge because they fragment irregularly under MS/MS conditions. Side-chain fluorinated polymers (SFPs), widely used in textiles and food packaging, degrade into a broad range of PFAS monomers and oligomers that seldom match known masses or fragmentation patterns [50]. As a result, traditional PFAS identification analogies (e.g., homologous series, CF_2_-based mass increments) may not apply. Ultra-high-resolution Fourier Transform Ion Cyclotron Resonance Mass Spectrometry (FT-ICR MS) combined with paired mass distance (PMD) network analysis has recently enabled sub-ppm identification of low-abundance PFAS transformation products. For instance, Zhang et al. [120] identified 142 suspect and 53 unknown PFAS transformation products during plasma treatment of PFOS, revealing competing chain-shortening and chain-lengthening mechanisms that would remain invisible under conventional Orbitrap workflows. Diagnostic fragment ions and neutral losses provide an additional line of evidence for assigning structures to unknown PFAS. Common PFAS-specific fragments include perfluoroalkyl ions such as CF_3_^−^, C_2_F_5_^−^, and C_3_F_7_^−^, while ether-containing PFAS can generate oxygenated fragments such as CF_3_O^−^ and C_2_F_5_O^−^ through cleavage of C–O bonds. Head-group losses such as CO_2_ and SO_3_ are frequently observed, whereas fluorosulfonate (FSO_3_^−^) is particularly diagnostic of sulfonated PFAS [107]. When library matching is unsuccessful, in silico fragmentation tools such as MetFrag, SIRIUS, and CFM-ID can further prioritize candidate structures by comparing experimental accurate-mass and MS/MS data with computationally predicted formulas, fragmentation trees, or spectra. Such computational approaches are particularly valuable for unknown PFAS lacking authentic standards or reference MS/MS spectra and can substantially reduce the candidate space requiring manual structural interpretation [107,121].

#### 3.1.3. Ion Mobility Spectrometry HRMS (IMS-HRMS)

Ion mobility spectrometry (IMS) coupled with HRMS adds an orthogonal, gas-phase separation that complements LC/GC by resolving ions based on mobility (drift time) and collision cross section (CCS) in addition to *m*/*z*. Because CCS reflects an ion’s size/shape, IMS-HRMS can separate co-eluting and isomeric PFAS (e.g., branched vs. linear, structural isomers) that are difficult or impossible to resolve by chromatography alone, thereby strengthening annotation confidence in complex matrices. Recent studies demonstrate that IMS-HRMS substantially improves PFAS annotation confidence, especially for ether PFAS, halogenated PFAS, and branched fluorotelomers [115]. Because CCS is an intrinsic molecular property, IMS-HRMS enables annotation of PFAS even when MS/MS fragmentation is ambiguous or incomplete. However, the technique faces limitations, including variability in CCS measurements across instruments, lack of standardization, and the scarcity of CCS values for many emerging PFAS [122]. IMS-HRMS also provides substantial advantages for non-target identification of previously unrecognized PFAS. An IMS-HRMS-based suspect and non-target workflow applied to wastewater identified 56 potential PFAS, of which 45 (>80%) were identified without reference standards; incorporation of CCS into homologous-series screening eliminated 63% of false-positive candidates, demonstrating the value of ion mobility information for increasing confidence in unknown-PFAS annotation in complex matrices [123].

### 3.2. Organofluorine Mass Balance Approaches

Organofluorine mass balance approaches including EOF, AOF, and TF have emerged as essential complementary tools because they quantify the fluorine content of entire PFAS mixtures, irrespective of molecular identity. These methods are critical for detecting unknown PFAS because they reveal the magnitude of PFAS contamination that remains undetected by targeted LC-MS/MS or even HRMS. Moreover, mass balance approaches provide unique insights into PFAS partitioning, sorption behavior, transformation potential, and treatability across complex matrices such as wastewater sludges, landfill leachates, biosolids, soils, sediments, and biological tissues [36,99]. Organofluorine mass balance determination relies on the premise that all PFAS contain at least one C-F bond and that these bonds are sufficiently stable to persist through extraction, digestion, and combustion, ultimately releasing fluoride ions that can be quantified with high accuracy. Thus, EOF, AOF, and TF collectively establish quantitative boundaries for the minimum and maximum fluorine load in a sample, enabling researchers to infer the presence of unidentified PFAS classes, quantify non-extractable PFAS fractions, and evaluate changes in PFAS composition after treatment processes such as adsorption, membrane separations, advanced oxidation, and thermal destruction [75].

Variability in fluorine recoveries across PFAS classes introduces additional uncertainty into mass balance interpretation. Reported recoveries for EOF and AOF methods differ substantially depending on extraction strategy (SPE-EOF vs. liquid EOF), PFAS polarity, chain length, and functional group (Table S4). Ultrashort-chain PFCAs (e.g., TFA, PFPrA; typically 0–40%), zwitterionic PFAS, and certain polymer-derived compounds often exhibit low or negligible recoveries (<20–40%), whereas long-chain PFAAs and hydrophobic PFAS show higher retention efficiencies (50–75%) [124,125]. These low recoveries arise primarily from mismatches between PFAS physicochemical properties and the extraction mechanism. Ultrashort-chain PFAS are highly polar and water-soluble and therefore exhibit weak retention on conventional SPE sorbents, whereas zwitterionic PFAS can display pH-dependent charge states and matrix interactions that reduce extraction efficiency. As summarized in SI2, these class-dependent recovery differences can substantially influence the apparent proportion of “unknown” fluorine in environmental samples. The study compared WAX, WCX, and HLB sorbents and found that sorbent and matrix chemistry strongly affected fluorine recovery. In particular, Ca^2+^ and Mg^2+^ significantly suppressed EOF with WCX, while HLB poorly retained ultrashort-chain PFAS. It also showed that inorganic fluorinated anions can be retained and therefore contaminate apparent organofluorine measurements [87].

Consequently, unidentified EOF or TF fractions may partly reflect methodological limitations rather than exclusively novel PFAS species, underscoring the need for cautious interpretation when using mass balance metrics to assess treatment performance or environmental burden. Koch et al. [126] proposed a top-down workflow in which OF/EOF is measured first to establish the overall organofluorine burden, followed by target analysis to determine the fraction explained by known compounds. The remaining unquantifiable organofluorine is then progressively investigated using complementary approaches such as the total oxidizable precursor (TOP) assay, suspect screening, and non-target screening. Another limitation is the presence of inorganic fluoroanions and other fluorinated compounds, such as pharmaceuticals. These compounds can cause analytical bias when calculating fractions. This bias cannot be accounted for by the EOF budget, and they can critically manipulate the balance closure [67].

#### 3.2.1. Extractable Organic Fluorine (EOF)

EOF has become one of the most widely used sum-parameter approaches for quantifying the unidentified fraction of PFAS in environmental matrices including water [17,127], sediment [75], biological samples [128,129] and commercial products [99]. EOF measures the total fluorine present in extractable organic compounds following solvent extraction or solid-phase extraction (SPE). Quantification is typically performed using combustion ion chromatography (CIC), where all fluorinated analytes are combusted to fluoride (F^−^) prior to ion chromatographic detection. High-resolution graphite furnace molecular absorption spectrometry (HR-GF-MAS) has also been applied as an alternative detection technique for the determination of EOF [31,130]. Fluorine mass balance analysis is achieved by pairing EOF measurements with targeted PFAS quantification, enabling assessment of the fraction of fluorine explained by identified PFAS versus the fraction attributable to unidentified or unknown organofluorine compounds [17]. Numerous studies demonstrate that targeted PFAS frequently account for only a minor portion of EOF, often less than one-third, indicating that a substantial proportion of fluorine in environmental samples resides in unidentified PFAS or other fluorinated organic species [36,131]. The first interlaboratory comparison of EOF methods demonstrated that while CIC-based EOF shows promising accuracy and robustness (85–109% recovery in fortified waters), between laboratory variability in unfortified environmental samples can reach 21–37%, underscoring the need for methodological harmonization [132]. In the same study, targeted PFAS explained as little as 2–60% of EOF across water and sludge matrices, and poor extraction efficiency was observed for ultrashort-chain acids such as TFA, highlighting both the magnitude of unidentified organofluorine and extraction-related uncertainty in mass balance interpretation [132]. Moreover, extraction recovery can change with sorbent type, pH, solvent composition, and matrix effects, all of which contribute to uncertainty in determining total organofluorine content [133].

For drinking water, studies show that 40–80% of EOF often remains unidentified even after screening for dozens of legacy and emerging PFAS [38]. In surface waters receiving wastewater effluent, the discrepancy is even larger; Bugsel et al. [47] reported that targeted PFAS accounted for less than 20% of EOF downstream of industrial discharges. Ruyle et al. [59] examined 73 wastewater effluents across the United States and found that regulated PFAS constituted only 7–8% of EOF, while the sum of 18 targeted PFAAs represented just 11–21% of total extractable organofluorine. These findings confirm that wastewater treatment systems release large, uncharacterized organofluorine loads into aquatic environments. EOF also plays a critical role in evaluating PFAS transformation processes. During oxidative or biological treatment, PFAS precursors can degrade into terminal PFAAs or generate new intermediate PFAS structures. While targeted analysis typically captures only the terminal acids, EOF reveals whether the total organofluorine load increases, decreases, or remains unchanged, providing mechanistic clues regarding transformation pathways [134]. For example, studies examining AFFF-contaminated groundwater undergoing remediation often report stable or slightly decreasing EOF even when targeted PFAAs increase, indicating precursor transformation rather than net PFAS removal [36]. In this sense, EOF is indispensable for distinguishing true PFAS degradation from mere structural rearrangement. EOF’s limitations are linked primarily to extraction efficiency and selectivity. Some PFAS classes such as ultrashort-chain acids, zwitterionic PFAS, fluorinated amines, and polymeric PFAS are poorly retained by SPE and can be significantly underestimated.

#### 3.2.2. Adsorbable Organic Fluorine (AOF)

Adsorbable organic fluorine (AOF) represents a related but more selective mass-balance parameter that focuses on fluorinated organic compounds capable of adsorbing to activated carbon. Whereas EOF captures all extractable organofluorine species, AOF isolates the subset that interacts strongly with hydrophobic carbon surfaces. A critical step in AOF analysis is the removal of inorganic fluoride, which otherwise contributes to the fluoride measured after combustion and can cause substantial positive bias. After sample loading, the activated carbon is therefore washed with a dilute nitrate solution (commonly NaNO_3_ or KNO_3_) to remove inorganic fluoride and other water-soluble inorganic fluorine species while retaining adsorbed organofluorine. However, this washing step introduces an important analytical trade-off because highly polar, ultrashort- and short-chain PFAS may also be desorbed and lost together with inorganic fluoride [133]. Forster et al. [135] optimized AOF by acidifying samples to pH < 2, loading them onto activated carbon, and applying a nitrate-based washing step before CIC for environmental waters and reported AOF recoveries of 46–112% for individual PFAS and 87% for a PFAS mixture, demonstrating broad but compound-dependent analytical recovery. This is critical because unknown PFAS may exhibit sorption behaviors distinct from well-studied long-chain PFAAs. For example, some ether PFAS show moderate hydrophobicity but strong electrostatic interactions, while many fluorinated precursors and intermediates exhibit weak sorption and therefore break through GAC systems early, even when long-chain PFAAs are efficiently removed. Like EOF, AOF is quantified via combustion ion chromatography, but the initial enrichment step relies on adsorption to activated carbon rather than SPE. As a result, AOF values are often lower than EOF, representing only the hydrophobic fraction. AOF values should not be interpreted simply as representing the hydrophobic organofluorine fraction because recovery also depends on molecular structure, chain length, functional group, adsorption efficiency, washing conditions, combustion efficiency, and sample matrix. However, this hydrophobic fraction is often of particular concern in treatment plants because it includes long-chain PFAAs, polyfluorinated precursors, polymer degradation fragments, and fluorinated surfactants that pose high bioaccumulative and toxicological risks [136].

Matrix composition represents an additional source of uncertainty because dissolved inorganic fluoride competes with the analytical objective of selectively measuring organically bound fluorine, while dissolved organic matter, ionic composition, suspended material, and other co-occurring constituents can potentially influence adsorption and recovery. Consequently, AOF values should be regarded as method- and matrix-dependent operational measurements rather than absolute measurements of total PFAS. Jones et al. [137] reported satisfactory recoveries for most tested surface-water and wastewater matrices, whereas landfill leachate exhibited markedly lower recoveries of only 34–39% due to breakthrough during activated-carbon adsorption, demonstrating that complex matrices can compromise AOF capture efficiency. Because many treatment systems rely on adsorption, AOF provides a more treatment-relevant metric than EOF and is increasingly being adopted in engineering assessments. In an advanced wastewater treatment plant, 44 targeted PFAS accounted for only 0.87–3.44% of AOF in filtered samples and 0.03–3.70% in unfiltered samples, demonstrating that the vast majority of carbon-adsorbable fluorine was not explained by conventional targeted PFAS analysis; however, this unidentified fraction may also include non-PFAS organofluorine compounds [138]. One challenge associated with AOF is the incomplete capture of polar PFAS, including short-chain acids and many newly developed replacement PFAS. These compounds contribute significantly to EOF but minimally to AOF, meaning that AOF cannot be used to infer total PFAS loads. Instead, AOF and EOF must be interpreted together to contextualize PFAS polarity, sorption behavior, and treatability across diverse systems [139,140,141].

#### 3.2.3. Total Fluorine (TF)

**Figure 4:**
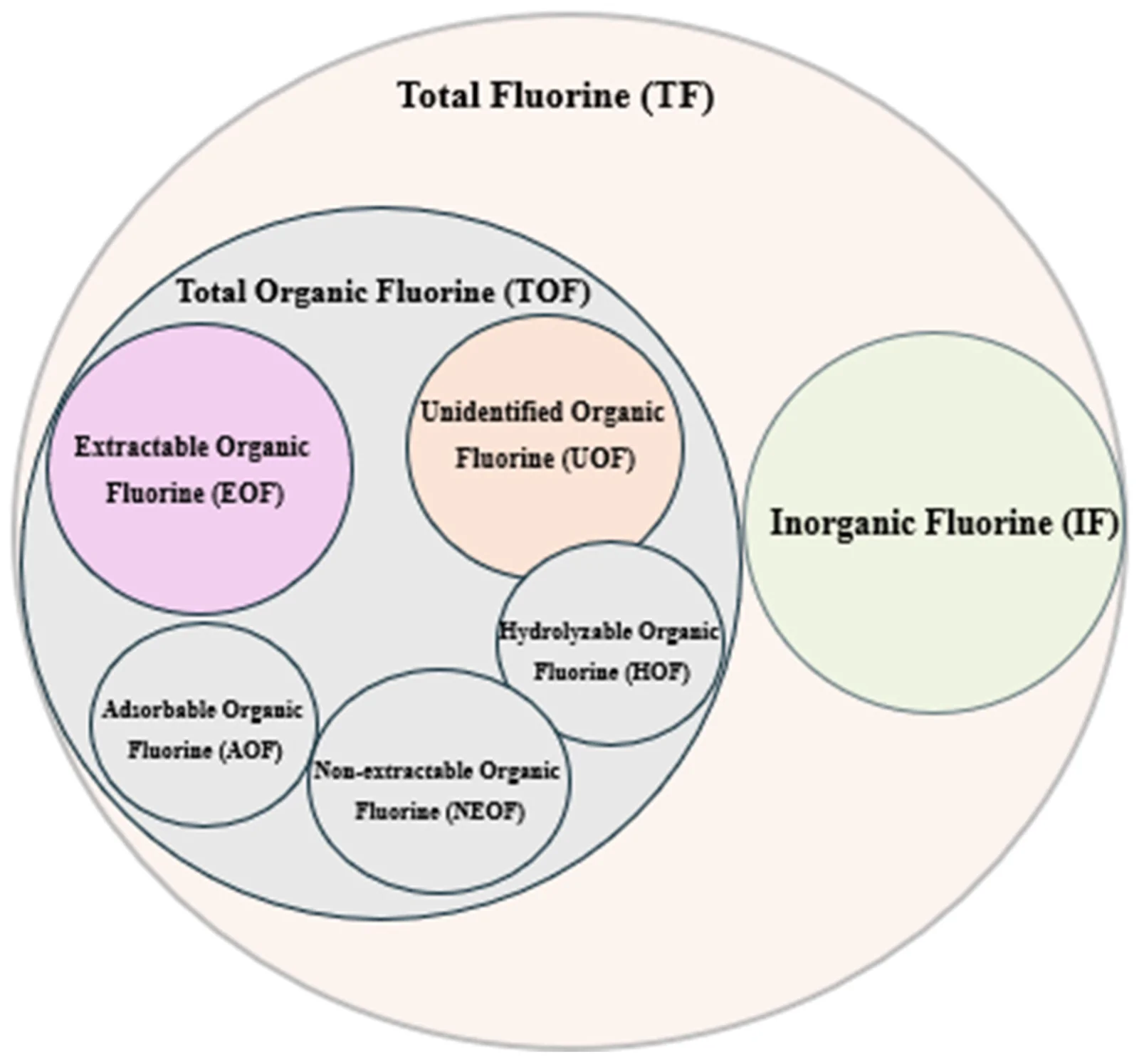
Overview of total fluorine (TF) analysis.

Total fluorine (TF) measurement provides the upper boundary for all fluorine present in a sample, encompassing inorganic fluorine (IF) and TOF (Equation (1)). Within the organic fluorine pool, operationally defined fractions may include EOF, AOF, hydrolysable organic fluorine (HOF), and unidentified organic fluorine (UOF), depending on the analytical workflow employed (Figure 4). As a mass balance tool, TF enables scientists to estimate the proportion of fluorine present as organic compounds (Equation (2)), as extractable organofluorine (EOF/TF), and as non-extractable organofluorine (Equation (3)) [142,143,144].

(1) TF=IF+TOF

(2) $$EOF=\sum F_{target}\;+\;UOF$$

(3) NEOF=TF−EOF−IF

TF is commonly determined by CIC, in which an unextracted sample is combusted at high temperature, fluorine-containing compounds are converted to hydrogen fluoride/fluoride and collected in an absorption solution, and fluoride is subsequently quantified by ion chromatography. Other fluorine-specific techniques can complement CIC, including particle-induced gamma-ray emission (PIGE) spectroscopy for nondestructive measurement of bulk fluorine, instrumental neutron activation analysis (INAA), and fluorine-19 nuclear magnetic resonance (^19^F-NMR) spectroscopy for organic fluorine characterization.

NEOF can constitute 50–95% of total fluorine in contaminated soils, indicating that the majority of PFAS-like substances in some systems are non-extractable and remain invisible to EOF-based methods [145,146]. These NEOF pools are of major environmental concern because they may undergo slow release over time, especially under pH changes, redox fluctuations, or thermal conditions, thereby contributing to long-term PFAS leaching. In engineered systems, TF-based mass balance is essential for evaluating treatment outcomes. For example, thermal treatment processes such as incineration, pyrolysis, or gasification can convert PFAS into inorganic fluoride (IF); tracking TF before and after treatment enables assessment of PFAS destruction efficiency vs. redistribution [147,148].

Hydrolyzable organically bound fluorine (HOF) is an emerging fluorine sum parameter designed to extend analytical coverage toward hydrolyzable, covalently bound organofluorine that may be poorly represented by conventional EOF measurements. The approach uses alkaline hydrolysis to release fluorinated moieties from hydrolyzable precursor structures, particularly side-chain fluorinated polymers (SFPs), followed by determination of the resulting organofluorine fraction using CIC [149]. Roesch et al. [149] demonstrated the utility of this approach in 39 consumer products comprising outdoor textiles, food-contact paper, carpets, and baking sheets. HOF concentrations ranged from 0.03 to 76.3 μg F/g, whereas EOF ranged from below the detection limit to 58.1 μg F/g. In textiles, hydrolytic extraction produced HOF concentrations of 1.38–76.3 μg F/g and, in some samples, values up to approximately tenfold higher than conventional EOF, demonstrating that hydrolysis can reveal organofluorine associated with hydrolyzable polymeric or precursor structures that conventional extraction may substantially underestimate [149]. The main limitation of TF is that it cannot differentiate between organic and inorganic fluorine without pre-fractionation and cannot distinguish specific PFAS classes. TF alone cannot infer PFAS risk because inorganic fluoride is much more abundant in many systems and does not pose the same environmental hazards. Nonetheless, TF is the only measurement capable of establishing the maximum possible PFAS contamination and is indispensable for mass-balance interpretation [145].

### 3.3. Precursor Oxidation Assays

The detection of unknown PFAS is complicated by the widespread occurrence of polyfluorinated precursors—compounds that are not terminal PFAAs but can transform into persistent PFAS through environmental or engineered oxidation processes. These compounds are intentionally oxidized because many unknown or analytically inaccessible precursors contain transformable non-perfluorinated moieties, whereas their perfluorinated carbon chains can be converted into more stable terminal PFAAs that are readily quantified by established targeted methods. These precursors include fluorotelomer-based surfactants, perfluoroalkyl sulfonamide derivatives, polyfluorinated phosphates (PAPs), zwitterionic PFAS, fluorinated ethers, and numerous proprietary mixtures used in industrial formulations [150,151]. Precursor oxidation assays therefore play a critical role in the detection of unknown PFAS because they provide an indirect measure of transformation-prone compounds, allowing researchers to infer the presence and abundance of precursor species that escape routine analysis. Among these, the TOP assay has emerged as the most widely used technique, although significant advances and limitations have shaped its interpretation in regulatory and engineering contexts [152].

#### 3.3.1. Total Oxidizable Precursor (TOP) Assay

The TOP assay is designed to convert polyfluorinated precursors into their corresponding terminal perfluoroalkyl acids using a strong oxidative system [152]. Typically, hydroxyl radicals (•OH) generated from persulfate activation cleave C-N, C-O, and partially fluorinated C-C linkages, ultimately producing stable perfluoroalkyl moieties that cannot be further oxidized under environmental or assay conditions [16]. Using the TOP assay on AFFF-impacted groundwater indicates that 25% of PFASs that can be oxidized to PFCAs remain unidentified [153]. Even widely used fluorotelomer-based PFAS may yield incomplete oxidation products, especially in matrices rich in organic matter, divalent cations, or radical scavengers [154]. However, despite its widespread use, the TOP assay possesses fundamental limitations that complicate its interpretation, particularly for unknown PFAS detection. Many modern PFAS including ether PFAS, branched PFAS, zwitterionic surfactants, fluorinated amines, cationic PFAS, and polymeric precursors do not fully oxidize under TOP assay conditions [131,155]. For example, perfluoroether carboxylic acids (PFEAs) such as HFPO-DA (GenX), ADONA, and related proprietary formulations are resistant to hydroxyl radical oxidation, reflecting their stabilized C-O-C linkage and electron-withdrawing fluorinated backbone. Similarly, polyfluorinated phosphoric acid esters (PAPs), commonly found in food contact materials and sewage sludge, undergo partial oxidation and yield complex intermediate fragments rather than predictable terminal PFAAs [156]. Ruyle et al. [59] showed that municipal wastewater effluent exhibited substantial precursor loads based on TOP-induced PFAA increases, yet these accounted for only 13–16% of EOF. This means that even after aggressive oxidation, the vast majority of fluorinated organics remain unidentified. Similar findings have been reported for landfill leachates, stormwater outfalls, and biosolids, where TOP-induced PFAA formation explained only a minor fraction of total PFAS or EOF [128,157]. Thus, the TOP assay provides valuable qualitative evidence for precursor presence but should not be interpreted as a comprehensive measure of unknown PFAS [158]. Another complication arises from the formation of ultrashort-chain PFAS during TOP oxidation. For example, TOP oxidation frequently yields TFA, perfluoropropionic acid (PFPrA), and PFBA, which are themselves environmentally persistent, mobile, and poorly removed by conventional water treatment processes [158]. Recent pilot scale work also shows that advanced oxidation processes (AOPs) can unintentionally transform previously non-detected or unknown longer-chain PFAS into measurable short-chain PFAAs, causing post-treatment increases in ΣPFAS of up to 95–340% in real groundwater matrices [159]. These findings emphasize that precursor-rich waters subjected to strong oxidative conditions may yield false indicators of PFAS “removal,” reinforcing the need to pair AOP testing with fluorine-based metrics such as AOF to capture unknown PFAS conversion. These ultrashort-chain products might originate either from precursor degradation or from oxidative scission of longer-chain PFAS. Consequently, the TOP assay may inadvertently create PFAS species that were not initially present in the environment, complicating mass balance interpretation and risk assessment. Matrix effects also influence TOP assay performance. Inorganic species such as bicarbonate and chloride can consume radicals or generate secondary reactive species that alter oxidation pathways, affecting the composition of post-TOP PFAA products [152,160]. Again, suspect/non-target screening further revealed a 346-fold post-dTOP increase in unsaturated PFCAs (dPFCAs), suggesting their formation as oxidation products of unidentified unsaturated PFAS precursors and demonstrating that oxidative precursor assays can reveal hidden PFAS classes not apparent before treatment [77]. These complexities illustrate why TOP results must be interpreted cautiously, especially for engineering applications such as treatment performance evaluation, residual PFAS forecasting, or mass balance modeling.

#### 3.3.2. TOP Combined with Bayesian Inference

As the limitations of the traditional TOP assay have become more apparent, new analytical frameworks have emerged to enhance precursor quantification. The integration of TOP with Bayesian inference (TOP with BI) represents a substantial methodological advancement. In this approach, the PFAA transformation profiles generated by the TOP assay are mathematically reconstructed using Bayesian modeling, which infers the most probable precursor composition based on known oxidation pathways, yields, and carbon chain scission patterns [161,162]. In the TOP + Bayesian inference framework, PFAA concentrations are measured before and after oxidation, and the post-TOP increases in individual PFAA homologues are combined with experimentally determined precursor-to-product oxidation yields [163]. This statistical framework or Bayesian modeling accounts for uncertainty in oxidation efficiency, incomplete radical exposure, and competing reaction pathways, producing more accurate estimates of precursor concentrations even when oxidation is incomplete. Recent applications of TOP with BI and related modeling frameworks at PFAS contaminated sites have reinforced these advantages. For example, Zweigle et al. [9] showed that even when dTOP substantially increased PFAAs in heavily contaminated soils, modeled precursor contributions still explained less than one-quarter of the total PFAS burden, indicating that large fractions of precursors and polymer-derived PFAS remain analytically invisible without NTA. Likewise, studies integrating TOP with comprehensive NTA in firefighting foams and refinery soils demonstrate that Bayesian or pattern-based reconstruction of precursor profiles can guide prioritization of high-risk precursors such as double bond perfluorinated alcohols, HFIP, and NTf_2_ for further toxicological and regulatory evaluation [164,165,166]. However, TOP with BI still cannot conclusively identify precursor structures. Its predictions rely on existing oxidation pathway knowledge, which is incomplete for many emerging PFAS, especially ether-based and polymer-derived species [167]. Furthermore, the presence of radical scavengers, sorbed PFAS, colloidal materials, or competing organic compounds may distort PFAA formation profiles and confound Bayesian reconstructions [163].

### 3.4. Fluorine-Specific Spectroscopic and Nuclear Methods

These techniques represent essential complementary tools for detecting unknown PFAS, especially in complex solids, industrial products, biological tissues, and matrices that are not easily amenable to chromatographic separation or mass spectrometric ionization [168]. Among these techniques, fluorine-19 nuclear magnetic resonance (^19^F-NMR) spectroscopy directly detects fluorine-containing molecular environments and can provide quantitative and structural information on organofluorine without requiring analyte ionization, whereas particle-induced gamma-ray emission (PIGE) spectroscopy measures the bulk total elemental fluorine signal and does not distinguish PFAS from other organic or inorganic fluorine species [169,170]. Their orthogonality to chromatographic and mass spectrometric tools makes them indispensable for source identification, bulk quantification, and elucidation of PFAS molecular environments that remain inaccessible through conventional techniques. While their inherent limitations prevent widespread application in trace-level water analysis, these methods are increasingly used to characterize unknown PFAS in polymeric materials, textiles, packaging, biosolids, sludges, and solid wastes where conventional extraction-based approaches capture only a fraction of total PFAS [171,172]. As PFAS regulations begin to shift toward total-fluorine-based criteria, fluorine-specific spectroscopic techniques are becoming central to both environmental monitoring and industrial compliance [173].

#### 3.4.1. ^19^F Nuclear Magnetic Resonance (^19^F-NMR)

^19^F nuclear magnetic resonance provides a uniquely powerful platform for direct detection of fluorinated functional groups in complex matrices because fluorine-19 is 100% naturally abundant, highly sensitive to its electronic environment, and exhibits a wide chemical shift range [174,175]. Unlike LC-MS/MS or HRMS, which rely heavily on ionization and retention behavior, ^19^F-NMR detects PFAS irrespective of polarity, molecular size, or ionization propensity, enabling the characterization of fluorinated compounds that remain completely invisible to chromatographic techniques [176,177]. This is particularly important for unknown PFAS, many of which are neutral, zwitterionic, polymeric, or highly hydrophobic, making them poorly amenable to traditional MS-based detection; additionally, ^19^F-NMR can quantify fluorinated compounds without requiring compound-specific reference standards, providing an important advantage for unknown PFAS for which authentic standards are unavailable [169]. Recent work has demonstrated that quantitative ^19^F-NMR can provide higher total PFAS estimates than both LC-HRMS and the TOP assay in complex wastewater matrices, indicating that traditional targeted and precursor oxidation approaches may substantially underreport total PFAS burdens [176]. In particular, ^19^F-NMR enabled detection of ultrashort-chain species such as TFA at concentrations several times higher than those captured by LC-MS, reinforcing its value as an orthogonal mass balance tool for unidentified fluorinated fractions. Mechanistically, ^19^F-NMR provides information about the chemical environment of CF_3_, CF_2_, and CF functional groups. Peaks corresponding to terminal CF_3_ groups, chain-internal CF_2_ units, and specialized moieties such as CF_2_-O or CF_2_-SO_2_R can be distinguished based on characteristic chemical shifts [178]. This capability allows researchers to differentiate between perfluorinated acids, sulfonates, ether-PFAS, fluorinated oligomers, and polymeric PFAS even when their full molecular structures and masses remain unknown. For example, PFAS polymers such as side-chain fluorinated polymers (SFPs), used widely in textiles and outdoor products, exhibit broad, distinct ^19^F-NMR spectral features associated with repeated CF_2_ units and polymeric backbones [9]. This enables their identification in solid consumer products and environmental residues, long before they degrade into quantifiable PFAS monomers. ^19^F-NMR has also been extended to biological matrices. Solid-state magic-angle spinning (MAS) ^19^F-NMR has successfully detected PFOA accumulation in murine liver, placenta, and adipose tissues, demonstrating the feasibility of direct PFAS quantification in biological tissues without extraction bias and highlighting potential bioaccumulation in compartments not traditionally associated with PFAS storage [179]. Such applications are particularly relevant for unknown PFAS exposure assessment, where compound-specific LC-MS methods may overlook emerging or structurally atypical PFAS. Beyond direct quantification, recent advances integrating machine learning with ^19^F-NMR spectral prediction have further enhanced its applicability to unknown PFAS discovery. Rao et al. [180] developed a neural-network-based model capable of predicting ^19^F chemical shifts with high accuracy, enabling improved peak assignment and structural clarification of previously unidentified PFAS in wastewater samples. This integration of predictive modeling with fluorine-specific spectroscopy represents a promising pathway toward reducing structural ambiguity in complex environmental mixtures. ^19^F-NMR is particularly useful for characterizing PFAS in biosolids, sludge, and biological tissues. Because these matrices contain large proportions of non-extractable PFAS, traditional SPE-based EOF workflows often underestimate total PFAS burdens. ^19^F-NMR, in contrast, detects bulk organofluorine independent of extractability, enabling the assessment of polymeric residues, persistent transformation intermediates, and unknown PFAS that strongly bind to organic matter or proteins [177]. Despite these strengths, ^19^F-NMR generally has lower sensitivity than LC-MS/MS or HRMS, although optimized preconcentration and noise-reduction approaches have achieved detection limits as low as 389 pg/L in environmental waters [169]. Spectral overlap in complex matrices can also complicate structural interpretation; however, characteristic ^19^F chemical shifts enable class-specific analysis without requiring authentic standards for every individual PFAS [181].

#### 3.4.2. Particle-Induced Gamma Ray Emission (PIGE)

Unlike mass spectrometry or NMR, PIGE relies on proton irradiation of a solid sample surface, which induces nuclear reactions that emit characteristic gamma rays proportional to fluorine content [182]. Because it does not require chemical extraction or digestion, PIGE provides rapid, non-destructive quantification of total fluorine load on the sample surface, making it invaluable for screening large numbers of industrial or consumer products [183]. PIGE has played a transformative role in revealing the presence of unknown PFAS in commercial goods [170]. Investigations using PIGE have identified significant fluorine levels in food contact papers, compostable materials, microwave popcorn bags, school uniforms, and firefighter turnout gear products that were not previously known to contain PFAS or were mislabeled as “fluorine-free” [172,184]. However, PIGE cannot distinguish between organic and inorganic fluorine without additional chemical extraction [185]. Moreover, because PIGE measures bulk fluorine rather than molecular identity, it cannot distinguish or quantify individual PFAS within complex mixtures; compound-specific techniques such as LC-MS/MS or HRMS are therefore required for subsequent identification. Tighe et al. [183] extended PIGE to drinking-water screening by concentrating PFAS on activated carbon felt and achieved detection limits below 50 ng/L as total fluorine using 2 L of water; increasing the sample volume to 10 L was projected to lower the detection limit to below 10 ng/L. Acidification to pH < 2 also minimized inorganic fluoride interference while retaining anionic PFAS. Recently, Robel et al. [186] demonstrated that the sum of targeted PFAS (ΣPFAS) represented only 0.2–14% of the TF, indicating that the majority of fluorine in these products was attributable to unidentified or non-targeted fluorinated compounds.

### 3.5. Thermal Desorption and Pyrolysis GC/MS

Thermal analytical techniques, particularly pyrolysis gas chromatography mass spectrometry (Py-GC/MS) and thermal desorption GC/MS (TD-GC/MS), provide a complementary tool for detecting unknown PFAS that are poorly amenable to liquid chromatography electrospray ionization workflows. In Py-GC/MS, samples are subjected to controlled high-temperature decomposition (typically 500–1000 °C), thermally cleaving polymer backbones and side chains into diagnostic fluorinated fragments that can be separated and identified via GC-MS. Characteristic pyrolysis products, including fluorotelomer fragments, perfluoroalkyl olefins, and short-chain PFAA, provide insight into precursor composition and degradation pathways. Muensterman et al. [187] used Py-GC/MS to differentiate PTFE films from textile layers treated with side-chain fluoropolymers in firefighter turnout gear. Targeted volatile and nonvolatile PFAS accounted for only 0.0016–6.7% of total fluorine, while the PTFE-containing moisture barrier contained up to 122,000 mg F/kg, demonstrating that substantial fluorine can reside in polymeric forms not represented by conventional targeted PFAS measurements. Recent studies applying Py-GC/MS to textiles, food-contact materials, and biosolids have demonstrated that large fractions of fluorine present as side-chain fluorinated polymers (SFPs) and fluoropolymer residues are not detectable by LC-MS/MS but can be structurally characterized through diagnostic thermal fragments, thereby revealing substantial pools of previously unrecognized organofluorine [188]. In contrast, TD-GC/MS focuses on the volatilization of intact semi-volatile and volatile PFAS prior to decomposition, making it particularly suitable for fluorotelomer alcohols (FTOHs), perfluoroalkyl sulfonamides, and other airborne or dust-associated precursors [189]. By thermally releasing these compounds from solid matrices without complete destruction, TD-GC/MS preserves structural information and enables detection of precursor PFAS that contribute to long-range transport and indoor exposure. Robbins et al. [190] developed a TD-GC-MS/MS method for 4:2, 6:2, 8:2, and 10:2 FTOHs emitted from consumer products, achieving instrument detection limits of 0.07–0.09 ng/tube. The method detected 6:2, 8:2, and 10:2 FTOHs from a stone cleaner/sealer and 8:2 and 10:2 FTOHs from mattress-pad products, illustrating its utility for characterizing volatile PFAS precursor emissions and potential indoor exposure pathways. Together, Py-GC/MS and TD-GC/MS expand the analytical window beyond ionizable PFAAs, bridging critical gaps in the detection of polymer-bound, volatile, and transformation-prone PFAS in complex matrices [190,191].

## 4. Key Analytical Challenges

The evaluation and treatment of unknown PFAS are fundamentally limited by analytical constraints that directly influence every stage of engineering practice, from system design and technology selection to regulatory compliance and long-term risk assessment. This unidentified fraction, sometimes referred to as “PFAS dark matter”, encompasses PFAS-related organofluorine that remains undetected or structurally uncharacterized by conventional targeted analytical methods [192]. Unknown PFAS display an extraordinary breadth of physicochemical properties, spanning ultrashort-chain acids, zwitterionic precursors, ether-PFAS, perfluorophosphates, cationic surfactants, and polymeric PFAS [193]. Such extreme structural heterogeneity makes detection inherently challenging and, in turn, undermines the development of robust treatment systems that can address the full PFAS spectrum. A comparative overview of the principal analytical techniques used for unknown PFAS detection is provided in Table 1.

Figure S1 further illustrates the complementary analytical coverage of targeted LC-MS/MS, HRMS-based suspect/non-target screening, TOP, EOF/AOF, and TF, highlighting the remaining blind spots and the broader coverage achievable through integrated multi-method analysis. Emerging sensor-based approaches, such as surface-enhanced Raman scattering (SERS), may further complement these established techniques by enabling rapid and highly sensitive PFAS screening, although broader validation across diverse PFAS classes and complex environmental matrices is still required [194].

Even sophisticated HRMS platforms capture only a small portion of this chemical space, primarily because structural annotation depends on reference standards, predictable fragmentation pathways, and reliable retention time indices. Isomeric complexity, co-elution, and incomplete fragmentation libraries further reduce the accuracy of suspect screening [24]. This incomplete coverage directly affects engineering decisions, as treatment systems are often designed, optimized, and validated based only on the small fraction of PFAS that can be measured reliably. Consequently, non-destructive systems such as GAC, IX, RO, and NF may appear highly effective when evaluated using targeted PFAS alone, even though they may allow substantial breakthroughs of unmonitored PFAS classes. Similarly, concentrates generated from membrane or IX systems may contain large quantities of unknown PFAS, complicating subsequent destruction steps and increasing disposal liabilities [195,196].

**Table 1:** Analytical coverage and limitations of current techniques for detecting and characterizing PFAS.

Technique	Coverage	Typical MDL	Limitation	Main Blind Spots	TRL	Ref.
LC-MS/MS (Targeted)	Known PFAS only	ng/L-pg/L	Cannot report unidentified PFAS; however, quantification of the known fraction provides the baseline for estimating unidentified PFAS through complementary fluorine mass-balance approaches	PFAS outside the predefined target list, particularly compounds lacking authentic reference standards; unknown transformation products, many precursors, polymeric PFAS, and other unidentified organofluorine remain unresolved	9	[126,197]
HRMS (NTA/SS)	Ionizable PFAS	ng/L	Limited reference standards, semi-quantitative	Poorly ionizing or non-ionizable PFAS, some neutral/volatile species better suited to GC-based analysis, polymeric/non-extractable PFAS, and features that cannot be confidently annotated because of limited standards and spectral libraries	7–8	[20,60]
LC/IMS-HRMS	Isomers and co-eluting PFAS	ng/L	Limited CCS libraries	PFAS lacking reliable CCS/reference data, poorly ionizing or non-ionizable species, and isomers with insufficient mobility separation; polymeric and non-extractable PFAS generally remain unresolved.	6–7	[91,198]
EOF (SPE-CIC)	Extractable organofluorine	µg/L F	No structural info	Non-extractable organofluorine and compounds poorly recovered by the selected extraction/sorbent system; provides no molecular identity and cannot establish whether measured organofluorine is PFAS	8	[133,146,157]
AOF	Carbon-adsorbable PFAS	µg/L F	Misses polar PFAS	Highly polar and ultrashort-chain organofluorines with poor activated-carbon retention; provides no molecular identity and may be affected by residual inorganic fluoride/background	7	[138,199]
TF	Total fluorine	µg/kg-mg/kg	Includes inorganic F	No molecular or class-specific information and no intrinsic distinction between organic and inorganic fluorine; high inorganic-fluoride backgrounds can obscure comparatively small organofluorine/PFAS contributions	8–9	[32,200]
TOP Assay	Oxidizable precursors	ng/L	Incomplete oxidation	Non-oxidizable PFAS and precursors that incompletely oxidize or form unmonitored products; precursor identity cannot generally be reconstructed uniquely, and ultrashort-chain products may be missed by subsequent targeted analysis	6–7	[152,201]
Py-GC/MS & TD- GC/MS	Polymeric PFAS	mg/kg	Semi-quantitative	Compound coverage depends strongly on volatility and thermal behavior; quantitative interpretation of polymeric PFAS is challenging, and thermally generated fragments may not uniquely identify the original PFAS/polymer structure	6	[187,202]
^19^F-NMR	Bulk fluorinated groups	ppm	High detection limits	Trace-level PFAS below NMR sensitivity and individual compounds obscured by overlapping resonances in complex mixtures; generally limited molecular speciation without sufficient spectral resolution/reference information	5–6	[169,181]
PIGE	Total surface fluorine	µg/cm^2^	No speciation	Cannot distinguish individual PFAS or organic from inorganic fluorine; measures bulk/surface fluorine and therefore provides little molecular or structural information	7	[170,185]

Fluorinated ethers, neutral PFAS, volatile PFAS, side-chain fluorinated polymers, oligomeric species, and perfluorophosphinic acids often ionize poorly or not at all under conventional electrospray ionization (ESI) conditions [192,203,204]. Some of these PFAS require alternative ionization techniques such as atmospheric pressure photoionization (APCI), while others may never be detected through MS due to their polymeric nature or high volatility [195,205]. Moreover, the inability to ionize many PFAS species makes inline MS-based monitoring infeasible, restricting the development of real-time feedback-controlled treatment systems [206]. During the TOP assay, many partially oxidized intermediates may form. Thermal desorption in HRMS can decompose fluorotelomer-based polymers into monomeric fragments, complicating structural interpretation. Similarly, electrochemical oxidation, plasma treatment, advanced oxidation processes, or thermal degradation pathways frequently convert complex PFAS into shorter chain species such as TFA that are more mobile and more difficult to remove [207,208]. These transformation dynamics blur the distinction between analytical artifacts and true treatment pathways. Although EOF and TF measurements offer broader coverage, they do not distinguish between PFAS classes, nor do they identify the proportion of PFAS that may be more toxic or mobile. AOF methods selectively capture hydrophobic PFAS but miss the highly mobile short-chain compounds that often dominate unknown fractions.

## 5. Future Directions in Unknown PFAS Detection

As research progresses, the emphasis must shift toward analytical approaches that expand molecular coverage, increase structural resolution, and integrate seamlessly with treatment technologies. Future HRMS workflows will rely on fully automated, AI-driven annotation systems capable of processing thousands of PFAS-like signals with minimal manual interpretation [209,210]. Machine learning (ML) models trained on PFAS-specific fragmentation, mass defects, retention-time patterns, and homologous series will greatly expand the detection of ether PFAS, zwitterionic PFAS, fluorotelomer precursors, and transformation intermediates [121,211]. IMS will expand through comprehensive CCS libraries and ML-based CCS prediction tools, enabling rapid annotation of isomers, isobars, and polymer-derived PFAS [212,213]. Coupling IMS with treatment reactors will help identify intermediates formed during oxidative, reductive, and thermal degradation, improving mechanistic understanding of PFAS transformation. EOF, AOF, TF, and emerging metrics like the organofluorine index (OFI) will play a central role in quantifying total PFAS burdens, including polymeric and ultrapolar PFAS. Future efforts should improve extraction efficiency, standardize workflows for solids, and integrate mass balance directly with HRMS to partition samples into known, suspect, and unknown PFAS fractions. Enhanced ^19^F-NMR (e.g., cryo-probe systems) will improve detection of polymeric PFAS, strongly sorbed PFAS, and solid-phase residues. Emerging fluorine-specific sensors and microfluidic detection platforms may enable rapid, field-deployable screening to complement HRMS (integrated systems). Future progress requires expanding global PFAS databases (CompTox, OECD PFAS lists, FluoroHub) with MS/MS spectra, CCS values, retention indexes, and fluorine mass balance datasets [195,214]. Open sharing of HRMS non-target data will accelerate discovery of recurring unknown PFAS patterns and support the establishment of new reference standards. As structural data remains limited, predictive toxicology tools (QSAR models, ML-based toxicity predictions, high content screening) will be essential to evaluate hazards of unknown PFAS. Future regulatory frameworks should transition from compound-by-compound limits toward class- or hazard-based approaches that better account for the cumulative burden and potential mixture effects of PFAS [4].

## 6. Conclusions

Unknown PFAS remain a major unidentified component of PFAS contamination, and reliance on targeted analysis alone can substantially underestimate the broader fluorinated chemical burden in environmental and engineered systems. The central finding of this review is that no single analytical method can adequately characterize this unidentified fraction; rather, complementary integration of fluorine mass balance, precursor assays, and structure-resolving suspect and non-target analyses is necessary to progressively constrain and identify unknown PFAS. Importantly, analytical uncertainty directly translates into uncertainty in exposure assessment and treatment performance, particularly when unidentified precursors and transformation products persist or form during remediation. Future progress should therefore prioritize improved detection of ultrashort-chain and poorly ionizable PFAS, greater fluorine mass-balance closure in real environmental matrices, standardized analytical workflows, and expanded spectral and CCS libraries. Ultimately, integrating these analytical advances with treatment evaluation and hazard-informed regulatory strategies will be essential to move PFAS management beyond a limited set of regulated compounds toward a more comprehensive assessment of the unidentified PFAS burden.

## Data Availability

The raw data supporting the conclusions of this article will be made available by the authors on request.

## Supplementary Materials

The following supporting information can be downloaded at: https://www.mdpi.com/article/10.3390/toxics14100924/s1, Table S1: Occurrence of unknown PFAS or unidentified organofluorine across environmental compartments; Table S2: The newly reported compounds across different industries; Table S3: Conventional and emerging pretreatment approaches for targeted and non-targeted PFAS analysis; Table S4: Fluorine recoveries for AOF and EOF methods; Table S5: Full names and abbreviations of PFAS (listed in Table S2); Figure S1: Conceptual comparison of the analytical coverage and remaining blind spots of complementary approaches for PFAS characterization. Dash lines indicate unresolved (analytical blind spots) analytical coverage.
