# Supplementary material for: Beyond Known PFAS: Emerging Analytical Strategies for Resolving the Hidden Fluorinated Chemical Space
Source: Toxics. 2026 Oct 19;14(10):924. doi: 10.3390/toxics14100924 (PMC13709915; doi:10.3390/toxics14100924)
Supplement: Supplementary file 1 [file toxics-14-00924-s001.zip › toxics-4596815-supplementary.pdf]

Supporting Information (SI)

for

Beyond Known PFAS: Emerging Analytical Strategies for Resolving the Hidden Fluorinated Chemical Space

Faisal Ahmed[1], Elif Yakamercan[1,2], Mahmut S. Ersan[1*]

[1]*Department of Civil Engineering, University of North Dakota, Grand Forks, ND 58202-8115, USA.*

[2] *Department of Environmental Engineering, Bursa Technical University, Yildirim 16310, Bursa, Turkiye*

Submitted to

Toxics

October 9, 2026

*Corresponding author: mahmut.ersan@und.edu (Mahmut S. Ersan)

Table S1. Occurrence of unknown PFAS or **unidentified organofluorine** across environmental compartments

Environmental Compartment	Primary Source	Methods	Unidentified Organofluorine or unknown PFAS	Reference
Atmosphere	WWTPs and landfills	Improved TOP assay applied to air samples; quantified ΔPFCAs after oxidation	ΣΔPFCAs = 2,188-12,718 pg/m^3; ultrashort-chain products contributed 43.8-97.8% of ΣΔ(C2-C12 PFCAs).	[1]
Municipal influent/effluent	WWTP	EOF (CIC) and targeted PFAS	One cited wastewater case: targeted PFAS accounted for only 0.5-1.3% of EOF in influent and 3.6% and 6.1% of EOF in effluent (i.e., the remainder effectively unidentified EOF).	[2]
Cross-matrix synthesis	water, wastewater, biota, sediments, air/dust, consumer products	Systematic review of TF/TOF/EOF/AOF/TOP studies	Examples compiled in the review: seawater 60-90% organofluorine unknown; wastewater only 10% of EOF explained by 73 PFAS (⇒ 90% unidentified); fish liver 83.8% total fluorine unidentified; human serum unexplained EOF as low as 24% in some studies.	[3]
Human samples	Norwegian adults	EOF tracking in individuals; discussion of unknown/precursor contributions	This paper section highlights that EOF can change independently of legacy PFAS and explicitly notes the role of transformation from "unknown precursor compounds," but the excerpted section does not provide a single numeric "unknown fraction" value.	[4]

River basin multimedia assessment	China: source waters, surface waters, sediments	Suspect and NTA (homologue- and fragment-based), CL assignment	Identified 88 PFAS total; 27 assigned CL4-5 (features supported by PFAS fragments but lacking definitive structures).	[5]
Drinking Water & Raw Water	DWTP effluent	Oxidative transformation of precursors	TFA, PFPrA, PFBA derivatives	[6,7]
	Chlorination/oxidation byproducts	Presence of ultrashort chain PFAS not included in targeted monitoring	Small mobile PFAS (C1- C3)	
			Unknown polar PFAS in EOF fraction	
Surface Waters (Dutch rivers)	Industrial discharge, urban inputs	Expanded target analysis, suspect & de novo non-target screening (SNTS) using LCMS/MS and LCtimsTOF MS (IMS-HRMS)	38 PFAS identified via expanded target list; 19 additional compounds annotated via SNTS (incl. unsaturated PFOS analogue, 6:2 $FtSO_2AoS$)	[8]
Surface Water	Urban runoff	Release of precursor mixtures	Ether PFAS (e.g., F-53B analogues)	[9]
	Industrial discharges	Polymer breakdown products	Novel fluorotelomer derivatives	
		Formation of ether/iodinated PFAS	Iodinated PFAS	
Groundwater (AFFF-impacted)	Firefighting training areas Military bases	Transformation of fluorotelomer sulfonamides, Leaching of unknown PFAS precursors	FTSA-derived products	[10]
			Unknown perfluorosulfonamido acids	
			Polymer degradation PFAS	
		Continuous input of precursors	Mobile organic fluorinated compounds (MOFCs)	[11]

Wastewater (Domestic & Industrial)	Consumer products Textile/leather industries	Transformation during biological treatment	Fluoropolymer impurities	
		Incomplete removal of mobile PFAS	Short-chain transformation products	
Municipal Wastewater	Domestic wastewater (pharmaceutical inputs, urban discharge)	Multisorbent SPE, LC-HRMS screening, targeted quantification, combustion ion chromatography (CIC) for fluorine mass balance	23 highly fluorinated PFAS detected at low sub ppt levels; 30 low-fluorinated pharmaceuticals contributed 28-42% of EOF; inorganic fluoroanions accounted for 7-19% of EOF	[12]
Wastewater (near Yangtze River)	Wastewater from a Fluorochemical Manufacturing Park	Quadrupole time-of-flight tandem mass spectrometry	37 novel PFAS detected	[13]
Landfill Leachate	Degradation of fluoropolymers in landfills	Slow transformation of side-chain fluorinated polymers	Polymeric PFAS degradation products	[14]
		Microbial degradation generating diverse PFAS	Unknown PFAA-precursors	
			Novel acidic PFAS	
Soils (Urban & Agricultural)	Biosolids	Sorption of unknown PFAS precursors,	Unidentified organofluorine in sediments	[10,15]
	Irrigation with contaminated water	Degradation of polymeric PFAS	Soil-bound precursors	
		Atmospheric deposition of volatile PFAS	Polymer degradation PFAS	
Biosolids / Sewage Sludge	Accumulation during wastewater treatment	Sorption of long-chain PFAS & precursors, Enrichment of	Polyfluoroalkyl phosphate diesters (PAPs)	[16]
			Side-chain fluoropolymer fragments	

		hydrophobic polymer-derived PFAS	Unidentified EOF	
Marine Mammals	Bioaccumulation through marine food webs	Biotransformation to unknown metabolites<br>Accumulation of lipophilic fluorinated compounds	Novel fluorotelomer sulfones<br>Lipophilic PFAS in blubber; Ether PFAS	[17,18]
Birds / Eggs	Trophic transfer from aquatic prey<br>Atmospheric deposition	Accumulation of precursors, Formation of unknown metabolites in eggs	Unidentified PFAS in bile/egg yolk, Novel long-chain PFAS	[14,19]
Humans	Blood/Plasma	LC-MS/MS (52 PFAS) and EOF via TOF-CIC	14-69% unidentified organofluorine	[20]
Humans / Domestic Animals	Drinking water<br>Diet (fish, meat, dairy)<br>Indoor dust	Biotransformation of precursor PFAS, Exposure to replacement PFAS not included in monitoring lists	Unidentified serum EOF, PFAS precursors in blood, Short-chain and polymer-based PFAS metabolites	[17,21]
Atmosphere (WWTP & Landfill Air)	Municipal WWTP emissions; landfill gas	Cartridge-based active air sampling, LC-MS/MS for neutral & ionizable PFAS, TOP assay for precursor quantification	Neutral PFAS dominated (Σn-PFAS up to 564,343 pg m^{-3}); unknown precursors accounted for 46.4-58.4 mol% of total PFAS (TOP-derived)	[1]
Soil	Near a fluorochemical production facility, Flanders, Belgium	Optimized dTOP assay with UHPLC-MS/MS target analysis and QToF-HRMS suspect/non-target screening (SS/NTS)	192 nmol/kg non-target precursors, representing 72% of PFAS burden; Vlietbos: 210 nmol/kg precursors (82%)	[22]

Human	Human serum (15 participants, North Carolina, USA)	Nontarget LC-IMS-MS; PIMMS; mass-defect and CCS/m/z filtering; suspect screening; MS/MS structural elucidation	2 novel PFAS structurally elucidated; 125 PFAS would have been missed by targeted analysis using 119 standards. Seven of eight novel PFAS/halogenoalkane features occurred in all 15 participants.	[23]
River water	PFAS-contaminated natural river water	FT-ICR MS with nontarget analysis and CF_2-based paired mass distance (PMD) network; suspect screening; LC-MS/MS	53 novel (unknown) PFOS TPs with RA >1%; 30 novel TPs after 30 min, of which 21 were relatively hydrophilic under the chromatographic conditions; 13 unknown modules and 40 unknown nodes detected. UV: 5 homologue groups disappeared and 10 new groups formed in the 15-PFAS mixture; 40 candidate unknown modules	[24]

Table S2. The newly reported compounds across different industries

Industrial sector	Matrix	Newly identified compounds	Confidence level*	Identification status	Contribution to total PFAS (newly reported PFAS/total identified PFAS)	Identification method	Ref.
Textile	Wastewater effluent	DTXSID30240816 and DTXSID90240817	3	Tentative	2/648	Non-target screening with HRMS	[25]
	Waterproof related samples and industrial sludge	BPAF C18, PFTrECA n=2, PFTeECA n=2, PFTeECA n=3, PFPeECA n=2, PFPeECA n=3	2	Tentative	36/94	Non-target screening with UPLC tandem quadrupole orbitrap mass spectrometry	[26]
		7:2:3 FTECA, 9:2:3 FTECA, 11:2:3 FTECA, 12:2:3 FTECA, 14:2:3 FTECA, 5:3 FTECA, 6:3 FTECA, 7:3 FTECA, 8:3 FTECA, 9:3 FTECA, 10:3 FTECA, 6:4 FTCA, 8:4 FTCA, 10:4 FTCA, m-8:4 FTCA, m-10:4 FTCA, m-12:4 FTCA, dH-PFHpA, dH-PFOA, dH-PFNA, dH-PFDA, dH-PFUdA, dH-PFDoA, H-6:1FTOH, H-8:1FTOH, H-10:1FTOH, H-	3	Tentative			

		12:11FTOH, H-14:1FTOH, 5:2 FTECA, 7:2 FTECA					
Paper products	Paper products samples	9:3 FTCA, and 8:2 FTOH-Sulfate	2	Tentative	7/160	UPLC/MS-MS and HPLC/QTOF-MS	[27]
		11:3 FTCA, 11:3 FTUCA, 13:3 FTUCA, 6:2 FTOH-Sulfate, 10:2 FTOH-Sulfate	3	Tentative			
AFFF	Soil (AFFF impacted field site)	6:2/6:2 FTSAm dimer and 6:2/8:2 FTSAm dimer	2	Tentative	2/124	Non-target screening with LC-QTOF-MS	[28]
		OPFCAs (n=5)	1	Confirmed	28/82	Suspect + non-target with UPLC-HRMS/MS	[29]
		H-OPFCAs (n=3-6)	2	Tentative			
Semiconductor	Wastewater	mH-PFCAs (m=3, n=1-3), mH-PFCAs (m=5, n=1-2), mH-PFCAs (m=6, n=1-3), mH-OPFCAs (m=2, n=3-4), mH-OPFCA (m=3, n=1), H-mOPFCA (m=2, n=4),	3	Tentative			

	2H-mOPFCA (m=2, n=3), PDAs (n-2,3), H-PAs (n=2-4), and mH-OPAs (m=2, n=2-4)					
	mOPFCAs (m=2, n=1,2)	3	Tentative			
	H-FBSA, FBSA-MeAm, FBSA-MeNO$_2$, FBSA-PrNO$_2$, H-FBSE(1), H-FBSE(2), H-E-FBSE, U-FBSE, U-E-FBSE, H-FBSAA, E-FBSAA(1), E-FBSAA(2), FBSAcAL(1), and FBSAcAL(2)	3	Tentative	29/83	UPHLC-MS	[30]
	E-FBSA(1), E-FBSA(2), FBSA-SO$_3$H, FBSA-Am, FBSA-diazene, E-FBSE(1), E-FBSE(2), E-FBSE(3), FBSE-SO$_3$H, FBSE-Am, H-FBSEE, FBSPrA, FBSAcAL hydrate, and FBSAcAL hemiacetal	2	Tentative			
	FBSAA	1	Confirmed			
Wastewater and lake	Not listed individually	1-3	Confirmed-tentative	22/102	LC-HRMS	[31]

Fluorochemical							
Electroplating	Electroplating sample, wastewater, and sludge	H-PFBA<br>OBS	3<br>2	Tentative<br>Tentative	2/91	Non-target screening with LC-HRMS	[32]
Firefighting Foams	Foam samples	BiFOSA , Pr-FOSA , U-PFOSNO, PrMeAmA-FOSA, PrMeAmG-FOSA, 6:2 FtTP-HPrAmEtA, 6:2FtTP-HPrAmFABA	3	Tentative	7/54	UPLC<br>TOP assay	[33]
Oil refinery	Soil	HFIP<br>hexafluoro-2,2-propanediolHFIP, C6-CUPFA	1<br>2	Confirmed<br>Tentative	2/34	LC-MS/MS and UPLC- MS/MS TOP assay in nontarget analysis	[34]
Crude oil	Surface water, sediment, crude oil, and sludge	HOPFLSA (n = 1)	3	Tentative	7/53	UHPLC-HRMS	[35]
	Surface water, sediment, and sludge	HPFLCA (n=1), HOPFLSA (n=4)	4	Tentative			
	Surface water	5:2 FTCA, 7:2 FTCA, and 11:2 FTCA					

Surface water and sediment	13:2 FTCA	

*PFAS-specific confidence scale;Level 1: confirmed with reference standard orindistinguishable from reference standard; Level 2: probable structure / strong MS/MS evidence; Level 3: tentative candidate structure; Level 4: unequivocal molecular formula; Level 5: exact mass/feature only. Level 1 are classified as confirmed, and those at other levels as tentative

Table S3. Conventional and emerging pretreatment approaches for targeted and non-targeted PFAS analysis

Pretreatment Method	Advantages	Application Scenarios	Ref.
Protein precipitation (PPT)	Rapid protein removal; minimizes matrix effects; simple solvent-based protocol; low-cost (salting-out option).	High-protein biological matrices (serum, plasma).	[36]
Digestion method	Complete protein breakdown; releases covalently/non-covalently bound PFAS; low reagent demand.	High-fat/high-protein tissues (liver, muscle, eggs).	[37]
Liquid-liquid extraction (LLE)	Simple setup; scalable; suitable for bulk liquid processing.	Liquid samples.	[38]

Solid-phase extraction (SPE)	High enrichment and cleanup efficiency; improves sensitivity; adaptable (offline/online automation).	Broad range of aqueous and biological matrices.	[39]
Solid-liquid extraction (SLE)	Effective for granular/solid matrices; compatible with ultrasound-assisted extraction.	Solid and semi-solid samples.	[40]
Ion-pair extraction (IPE)	Enhances recovery of strongly ionized PFAS; improves extraction from complex matrices.	Matrices rich in proteins, lipids, humic substances.	[41]
QuEChERS method	Rapid, low-cost, reduced solvent use; suitable for high-throughput multi-residue screening.	Food, biological fluids, sediments.	[42]
Weak Anion Exchange (WAX-SPE)	Dual retention (reversed-phase with anion exchange); improved selectivity for anionic PFAS; effective for short- and long-chain species.	Drinking water, wastewater, air, serum.	[43]
Solid Phase Microextraction (SPME)	Solvent-free; minimal sample handling; suitable for volatile/semi-volatile PFAS.	Water, air, volatile PFAS precursors.	[44]
Accelerated Solvent Extraction (ASE)	Automated high-efficiency extraction under elevated temperature/pressure; strong recovery from solids.	Soil, sediment, biosolids, sludge.	[45]
Supercritical Fluid Extraction (SFE)	Reduced solvent consumption; effective for hydrophobic and polymeric PFAS.	Solid matrices, polymer-containing materials.	[46]

Sorbent-assisted adsorption-pyrolysis screening	Solvent-free enrichment; enables rapid screening of adsorbed PFAS via thermal decomposition GC/MS.	Aqueous screening, environmental monitoring.	[47]

Table S4. Fluorine recoveries for AOF and EOF methods

	References	[48]		[49]	[50]		[51]	[52]	[53]	[54]						[55]	
	**Methods**	SP-EOF	AOF	AOF	L-EOF	SP-EOF	AOF	AOF	L-EOF	SP-EOF	L-EOF	L-EOF	SP-EOF	SP-EOF	L-EOF	SP-EOF	AOF
**PERFLUOROALKYL CARBOXYLIC ACIDS (PFCAs)**	**TFA**			0				2								21	10
	**MMF**	72															
	**PFPrA**		68	0												33	25
	**PFBA**		64	52			40	42	70						60		70
	**PFPeA**								40						55		
	**PFHxA**								74						48		
	**PFHpA**								67			55			58		
	**PFOA**			64					67			48			50		
	**PFNA**			47	74.4				63			46		70	45		
	**PFDA**			41					68			48			52		
	**PFUnDA**								69		74	41			52		
	**PFDoDA**		69								72	50	60	58	40		
	**PFTrDA**									45	74	48	25	26	35		
	**PFTeDA**								68			42	6	10	18		
	**PFHxDA**											25	1	2	40		
	**PFOcDA**											28	0	1			
**PERFLUOROALKYL SULFONIC ACIDS (PFSAs)**	**TMSA**			0												52	18
	**PFBS**								20								70
	**PFPeS**											62			50		
	**PFHxS**											60					
	**PFHpS**											63					
	**PFOS**			64				65	57								
	**PFNS**														50		
	**PFDS**									72	74	68			70		
	**PFDoDS**											38	10	20	55		
**FLUOROTELOMER CARBOXYLIC ACIDS (FTCAs)**	**3:3 FTCA**												60		40		
	**5:3 FTCA**												35		50		
	**6:2 FTUCA**											52	50		40		
	**7:3 FTCA**											38	55		30		
	**8:2 FTUCA**											21		70	20		

	**8:2 FTCA**																
	**10:2 FTUCA**											21			20		
**FLUOROTELOMER SULFONIC ACIDS (FTSAs)**	**6:2 FTS**			63							70						
**POLY- and PERFLUOROETHER CARBOXYLIC ACIDS (PFECAs)**	**PFMOAA**						19										
	**PMPA**		46				21										
	**PFO2HxA**		73				60										
	**PFO2HxA**		73														
	**PEPA**		65				58										
	**HFPO-DA**									62	58						
	**PFO4DA**																
	**ADONA**											56			58		
**POLY- and PERFLUOROETHER SULFONATIC ACIDS (PFESAs)**	**Hydrolyzed PSDA**		70														
	**PS Acid**		68														
**CHLORINATED PERFLUOROETHERALKANE SULFONIC ACIDS**	**9Cl-PF3ONS**														60		
**POLY and PERFLUOROALKYL SULFONAMIDES and SULFONAMIDO SUBSTANCES (FASAs)**	**FBSA**														50		
	**MeFBSA**														40		
	**FHxSA**											42			45		
	**MeFHxSA**											43			35		
	**FOSA**				68.3				73		72				32		
	**MeFOSA**											26			30		
	**EtFOSA**				52							5					
	**FOSAA**								51								
	**MeFOSE**											1	35	50	25		
	**MeFOSAA**											62			60		
	**N-EtFOSE**								34		20	0	60	55	20		
	**EtFOSAA**											60			62		
**PERFLUOROALKANE SULFONYL FLUORIDE (PASF)**	**PBSF**			16													
	**POSF**			26													
	**4:2 FTOH**	48	44														
	**10:2 FTOH**	55	50														
	**PFHxPA**											55			58		

POLY AND PERFLUOROALKYL PHOSPHONIC ACIDS AND FLUOROTELOMER PHOSPHATES	**6:2 monoPAP**										30		0	40			
	**PFOPA**											5	0	42			
	**8:2 monoPAP**										58		0	45			
	**PFDPA**												1	36			
	**10:2 monoPAP**														35		
	**6:2 diPAP**									45			40				
	**6:2/8:2 diPAP**									8			5	15			
	**8:2 diPAP**									5			0	1	70		
	**10:2 diPAP**												0	1	50		
	**diSAmPAP**											46					
	**6:6 PFPi**											36	58	65	48		
	**6:8 PFPi**											12	15	15			
	**8:8 PFPi**											10	0	2	10		
ZWITTERIONS	**N-AP-FHxSA**						53										
	**N-TAMP-FHXSA**						63										

.

Table S5. Full names and abbreviations of PFAS (listed in **Table S2**)

Abbreviations	Formula	Full name
11:2 FTCA	$C_{13}H_3F_{23}O_2$	11:2 Fluorotelomer Carboxylic Acid
7:2 FTCA	$C_9H_3F_{15}O_2$	7:2 Fluorotelomer Carboxylic Acid
5:2 FTCA	$C_7H_3F_{11}O_2$	5:2 Fluorotelomer Carboxylic Acid
13:2 FTCA	$C_{15}H_3F_{27}O_2$	13:2 Fluorotelomer Carboxylic Acid
HPFLCA (n = 1)	$C_8H_2F_{14}O_2$	1:1 Fluorotelomer carboxylic acid
HOPFLSA (n=4)	$C_6H_2F_{12}O_4S$	4-hydroxyhexafluoroperfluoroalkyl sulfonic acid
HOPFLSA (n = 1)	$C_3H_2F_6O_4S$	1-hydroxytrifluoroperfluoroalkyl sulfonic acid
HFIP	$C_3H_2F_6O$	hexafluoro-2,2- propanediol
C6-CUPFA	C_6HF_9O	C6-cyclic-unsaturated perfluoroalkyl alcohol
HFIP	$C_3H_2F_6O$	hexafluoroiso- propanol
H-PFBA	$C_4H_2F_6O_2$	Hydro-substituted perfluorobutanoic acid
OBS	$C_{15}H_4F_{17}O_4S$	Sodium p-perfluorous nonenoxybenzene sulfonate
FBSAA	$C_6H_4F_9NO_4S$	(1,1,2,2,3,3,4,4,4-nonafluorobutane-1-sulfonamido)acetic acid
E-FBSA (isomer 1,2)	$C_4H_2F_9NO_3S$	Perfluorobutyl ether sulfonamide
FBSA-SO_3H	$C_4HF_9S_2O_5N$	(1,1,2,2,3,3,4,4,4-nonafluorobutane-1-sulfonyl)sulfamic acid
FBSA-Am	$C_5H_3F_9N_2O_3S$	N-carbamoyl-1,1,2,2,3,3,4,4,4-nonafluorobutane-1-sulfonamide
FBSA-diazene	$C_5H_4F_9N_3O_2S$	N-(diazenylmethyl)-1,1,2,2,3,3,4,4,4-nonafluorobutane-1-sulfonamide
E-FBSE (isomer 1,2,3)	$C_6H7F_8NO_3S$	Perfluorobutyl ether sulfonamido ethanol
FBSE-SO_3H	$C_6H_6F_9NO_6S_2$	N-sulfo perfluorobutane sulfonamido ethanol
FBSE-Am	$C_7H_7F_9N_2O_4S$	N-carbamoyl perfluorobutane sulfonamido ethanol
H-FBSEE	$C_8H_{11}F_8NO_4S$	Hydro-substituted N,N-bis(2-hydroxyethyl) perfluorobutanesulfonamide
FBSPrA	$C_7H_6F_9NO_4S$	Perfluorobutane sulfonamido propanoic acid
FBSAcAL hydrate	$C_6H_6F_9NO_4S$	N-(2,2-dihydroxyethyl)-1,1,2,2,3,3,4,4,4-nonafluorobutane-1-sulfonamide
FBSAcAL hemiacetal	$C_7H_8F_9NO_4S$	1,1,2,2,3,3,4,4,4-nonafluoro-N-(2-hydroxy-2-methoxyethyl)butane-1-sulfonamide
H-FBSA	$C_4H_2F_9NO_2S$	1,1,2,2,3,3,4,4,4-nonafluorobutane-1-sulfonamide
FBSA-MeAm	$C_6H_5F_9N_2O_3S$	2-(1,1,2,2,3,3,4,4,4-nonafluorobutane-1-sulfonamido)acetamide

FBSA-MeNO2	$C_5H_3F_9N_2O_4S$	1,1,2,2,3,3,4,4,4-nonafluoro-N-(nitromethyl)butane-1-sulfonamide
FBSA-PrNO2	$C_7H_7F_9N_2O_4S$	1,1,2,2,3,3,4,4,4-nonafluoro-N-(3-nitropropyl)butane-1-sulfonamide
H-FBSE (isomer 1,2)	$C_6H_7F_8NO_3S$	Hydro-substituted perfluorobutane sulfonamido ethanol
H-E-FBSE	$C_6H_7F_8NO_4S$	1,1,2,2-tetrafluoro-N-(2-hydroxyethyl)-2-(1,1,2,2-tetrafluoroethoxy)ethane-1-sulfonamide
U-FBSE	$C_6H_6F_7NO_3S$	1,1,2,2,3,4,4-heptafluoro-N-(2-hydroxyethyl)but-3-ene-1-sulfonamide
U-E-FBSE	$C_6H_6F_7NO_4S$	1,1-difluoro-N-(2-hydroxyethyl)-1-[(1,1,2,3,3-pentafluoroprop-2-en-1-yl)oxy]methanesulfonamide
H-FBSAA	$C_6H_5F_8NO_4S$	[(1,1,2,2,3,3,4,4-octafluorobutane-1-sulfonyl)amino]acetic acid
E-FBSAA (isomer 1,2)	$C_6H_4F_9NO_5S$	Perfluorobutyl ether sulfonamido acetic acid
FBSAcAL (isomer 1)	$C_6H_4F_9NO_3S$	Perfluorobutane sulfonamido acetaldehyde, enol tautomer
FBSAcAL (isomer 2)	$C_6H_4F_9NO_3S$	Perfluorobutane sulfonamido acetaldehyde, keto tautomer
mH-PFCA	$C_6H_6F_6O_2$	Hydrosubstituted perfluoroalkyl carboxylic acid
OPFCA	$C_6HF_{11}O_3$	ether-inserted PFCA
9:3 FTCA	$C_{12}H_5F_{19}O_2$	9:3 Fluorotelomer carboxylic acid
11:3 FTCA	$C_{14}H_5F_{23}O_2$	11:3 Fluorotelomer carboxylic acid
11:3 FTUCA	$C_{14}H_3F_{23}O_2$	11:3 Fluorotelomer unsaturated carboxylic acid
13:3 FTUCA	$C_{16}H_3F_{27}O_2$	13:3 Fluorotelomer unsaturated carboxylic acid
X:6 FTOH-Sulfate	$C_8H_5F_{13}O_4S$	X:6 Fluorotelomer alcohol sulfate
X:8 FTOH-Sulfate	$C_{10}H_5F_{17}O_4S$	X:8 Fluorotelomer alcohol sulfate
X:10 FTOH-Sulfate	$C_{12}H_5F_{21}O_4S$	X:10 Fluorotelomer alcohol sulfate
7:2:3 FTECA	$C_{12}H_9F_{15}O_3$	2-(pentadecafluoroheptyl)-1,3-dioxolane-2-ethanol
9:2:3 FTECA	$C_{14}H_9F_{19}O_3$	2-(nonadecafluorononyl)-1,3-dioxolane-2-ethanol.
11:2:3 FTECA	$C_{16}H_9F_{23}O_3$	2-(tricosafuoroundecyl)-1,3-dioxolane-2-ethanol.
12:2:3 FTECA	$C_{17}H_9F_{25}O_3$	2-(pentacosafuorododecyl)-1,3-dioxolane-2-ethanol.
14:2:3 FTECA	$C_{19}H_9F_{29}O_3$	2-(nonacosafuorotetradecyl)-1,3-dioxolane-2-ethanol
5:3 FTECA	$C_8H_5F_{11}O_3$	2-(Hendecafluoropentyl)-1,3-dioxolane-2-ethanol
6:3 FTECA	$C_9H_5F_{13}O_3$	2-(Tridecafluorohexyl)-1,3-dioxolane-2-ethanol
7:3 FTECA	$C_{10}H_5F_{15}O_3$	2-(Pentadecafluoroheptyl)-1,3-dioxolane-2-ethanol
8:3 FTECA	$C_{11}H_5F_{17}O_3$	2-(Heptadecafluorooctyl)-1,3-dioxolane-2-ethanol

9:3 FTE-CA	$C_{12}H_5F_{19}O_3$	2-(Nonadecafluorononyl)-1,3-dioxolane-2-ethanol
10:3 FTECA	$C13H_5F_{21}O_3$	2-(Heneicosafluorodecyl)-1,3-dioxolane-2-ethanol
6:4 FTCA	$C_{10}H_7F_{13}O_2$	2-(Perfluorohexyl)ethyl acetate)
8:4 FTCA	$C_{12}H_7F_{17}O_2$	2-(Perfluorooctyl)ethyl acrylate
10:4 FTCA	$C_{14}H_7F_{21}O_2$	2-(Perfluorodecyl)ethyl acrylate
m-8:4 FTCA	$C_{12}H_7F_{17}O_2$	2-(Perfluorooctyl)ethyl acetate
m-10:4 FTCA	$C_{14}H_7F_{21}O_2$	2-(Perfluorodecyl)ethyl acetate veya 1H,1H,2H,2H-Perfluorododecyl acetate
m-12:4 FTCA	$C_{16}H_7F_{25}O_2$	2-(Perfluorododecyl)ethyl acetate
dH-PFHpA	$C_7H_2F_{10}O_2$	Decafluoroheptane-2,4-dione
dH-PFOA	$C_8H_2F_{12}O_2$	2H-Perfluoro-2-octenoic acid
dH-PFNA	$C_9H_2F_{14}O_2$	7:2 Fluorotelomer unsaturated carboxylic acid
dH-PFDA	$C_{10}H_2F_{16}O_2$	8:2 Fluorotelomer unsaturated carboxylic acid
dH-PFUdA	$C_{11}H_2F_{18}O_2$	9:2 Fluorotelomer unsaturated carboxylic acid
dH-PFDoA	$C_{12}H_2F_{20}O_2$	10:2 Fluorotelomer unsaturated carboxylic acid
H-6:1FTOH	$C_7H_4F_{12}O$	Dodecafluoroheptanol
H-8:1FTOH	$C_9H_4F_{16}O$	Hexadecafluorononanol
H-10:1FTOH	$C_{11}H_4F_{20}O$	Eicosafluoroundecanol
H-12:11FTOH	$C_{13}H_4F_{24}O$	Tetracosafluorotridecanol
H-14:1FTOH	$C_{15}H_4F_{28}O$	Octacosafuoropentadecanol
5:2 FTECA	$C_7H_3F_{11}O_3$	Methyl perfluoro(2-methyl-3-oxahexanoate)
7:2 FTECA	$C_9H_3F_{15}O_3$	Methyl perfluoro(2-methyl-3-oxaoctanoate)
BPAF C18	$C_{18}H_{10}F_{12}O_2$	4,4'-Bis(2-hydroxyhexafluoroisopropyl)biphenyl
PFTrECA n=2	$C_{11}HF_{21}O_5$	Perfluoro-2,5,8-trimethyl-3,6,9-trioxadodecanoic acid
PFTeE-CA n=2	$C_{14}HF_{27}O_6$	Perfluoro-3,6,9,12-tetraoxapentadecanoic acid
PFTeECA n=3	$C_{15}HF_{29}O_6$	Perfluoro-2,5,8,11-tetramethyl-3,6,9,12-tetraoxapentadecanoic acid
PFPeECA n=2	$C_{17}HF_{33}O_7$	Perfluoro-3,6,9,12,15-pentaoxaoctadecanoic acid
PFPeECA n=3	$C_{18}HF_{35}O_7$	Perfluoro-2,5,8,11,14-pentamethyl-3,6,9,12,15-pentaoxaoctadecanoic acid
DTXSID30240816	$C_{18}H_{12}F_8N_2O_3$	2,6-Difluoro-N-(((2-methyl- 4-(2,2,2-trifluoro-1-hydroxy-1-(trifluoromethyl)ethyl)phenyl)amino) carbonyl)benzamide

DTXSID90240817	$C_{19}H_{14}F_8N_2O_3$	2,6-Difluoro-N-{[4- (1,1,1,3,3,3-hexafluoro-2-hydroxypropan-2-yl)-2,5-dimethylphenyl]car- bamoyl}benzamide
6:2/6:2 FTSAm dimer	$C_{16}H_9F_{26}NO_4S_2$	6:2/6:2 Fluorotelomer sulfonamide dimer
6:2/8:2 FTSAm dimer	$C_{18}H_9F_{30}NO_4S_2$	6:2/8:2 Fluorotelomer sulfonamide dimer
OPFCAs (n=5)	$C_6HF_{11}O_3$	Ether-substituted perfluoroalkyl carboxylic acid, perfluorinated chain length n=5
H-OPFCAs (n=3)	$C_5H_2F_8O_3$	Hydro-substituted ether-substituted perfluoroalkyl carboxylic acid, n=3
H-OPFCAs (n=4)	$C_6H_2F_{10}O_3$	Hydro-substituted ether-substituted perfluoroalkyl carboxylic acid, n=4
H-OPFCAs (n=5)	$C_7H_2F_{12}O_3$	Hydro-substituted ether-substituted perfluoroalkyl carboxylic acid, n=5
H-OPFCAs (n=6)	$C_8H_2F_{14}O_3$	Hydro-substituted ether-substituted perfluoroalkyl carboxylic acid, n=6
mH-PFCAs (m=3, n=1)	$C_4H_4F_4O_2$	Trihydrogen-substituted perfluorobutanoic acid
mH-PFCAs (m=3, n=2)	$C_5H_4F_6O_2$	Trihydrogen-substituted perfluoropentanoic acid
mH-PFCAs (m=3, n=3)	$C_6H_4F_8O_2$	Trihydrogen-substituted perfluorohexanoic acid
mH-PFCAs (m=5, n=1)	$C_6H_6F_6O_2$	Pentahydrogen-substituted perfluorohexanoic acid
mH-PFCAs (m=5, n=2)	$C_7H_6F_8O_2$	Pentahydrogen-substituted perfluoroheptanoic acid
mOPFCAs (m=2, n=1)	$C_4HF_5O_4$	Ether-substituted perfluoroalkyl carboxylic acid (Dioxa-PFCA)
mOPFCAs (m=2, n=2)	$C_5HF_7O_4$	Ether-substituted perfluoroalkyl carboxylic acid (Dioxa-PFCA)
mH-PFCAs (m=6, n=1)	$C_6H_7F_5O_2$	Hexahydrogen-substituted perfluorohexanoic acid
mH-PFCAs (m=6, n=2)	$C_7H_7F_7O_2$	Hexahydrogen-substituted perfluoroheptanoic acid
mH-PFCAs (m=6, n=3)	$C_8H_7F_9O_2$	Hexahydrogen-substituted perfluorooctanoic acid
mH-OPFCAs (m=2, n=3)	$C_5H_3F_7O_3$	3-[difluoro(perfluoromethoxy)methoxy]-2,2-difluoropropanoic acid
mH-OPFCAs (m=2, n=4)	$C_6H_3F_9O_3$	2,2,3,3-tetrafluoro-4-[difluoro(trifluoromethoxy)methoxy]butanoic acid
mH-OPFCA (m=3, n=1)	$C_4H_4F_4O_3$	[difluoro(methoxy)methoxy]methoxyacetic acid
H-mOPFCA (m=2, n=4)	$C_6H_2F_{10}O_4$	2,2-difluoro-2-[difluoro[fluoro(trifluoromethoxy)methoxy]methoxy]acetic acid
2H-mOPFCA (m=2,n=3)	$C_5H_3F_7O_4$	2,2-difluoro-2-[fluoro[fluoro(trifluoromethoxy)methoxy]methoxy]acetic acid
PDAs (n-2)	$C_3H_2F_6O_2$	Perfluoroalkyl double alcohol, n=2
PDAs (n-3)	$C_4H_2F_8O_2$	Perfluoroalkyl double alcohol, n=3
H-PAs (n=2)	$C_3H_2F_6O$	Hydro-substituted perfluoroalkyl alcohol, n=2
H-PAs (n=3)	$C_4H_2F_8O$	Hydro-substituted perfluoroalkyl alcohol, n=3
H-PAs (n=4)	$C_5H_2F_{10}O$	Hydro-substituted perfluoroalkyl alcohol, n=4
mH-OPAs (m=2, n=2)	$C_3H_3F_5O_2$	Multiple (m=2) H-substituted, ether-substituted perfluoroalkyl alcohol, n=2
mH-OPAs (m=2, n=3)	$C_4H_3F_7O_2$	Multiple (m=2) H-substituted, ether-substituted perfluoroalkyl alcohol, n=3
mH-OPAs (m=2, n=4)	$C_5H_3F_9O_2$	Multiple (m=2) H-substituted, ether-substituted perfluoroalkyl alcohol, n=4
BiFOSA	$C_{16}F_{34}NO_4S_2$	bis- (perfluorooctyl sulfonyl)amine
Pr-FOSA	$C_{11}H_8F_{17}NO_2S$	perfluorooctane sulfona-mide propene
U-PFOSNO	$C_{13}H_{11}F_{17}N_2O_3S$	unsaturated perfluorooctane sulfonamido amine oxide
PrMeAmA- FOSA	$C_{13}H_{12}F_{17}N_2O_3S$	perfluorooctane sulfonamido propyl methyl amino formamide
PrMeAmG-FOSA	$C_{16}H_{23}F_{17}N_2O_4S$	perfluorooctane sulfonamido propyl dimethyl amino propylene glycol
6:2 FtTP-HPrAmEtA	$C_{14}H_{15}F_{13}NO_4S$	6:2 fluorote-lomer thio- ether propanoyl hydroxy isopropyl amino ethanoic acid
6:2FtTP-HPrAmFABA	$C_{17}H_{20}F_{13}N_2O_5S$	6:2 fluorote-lomer thioether propanoyl hydroxy isopropyl amino formylamino isobu-tyric acid

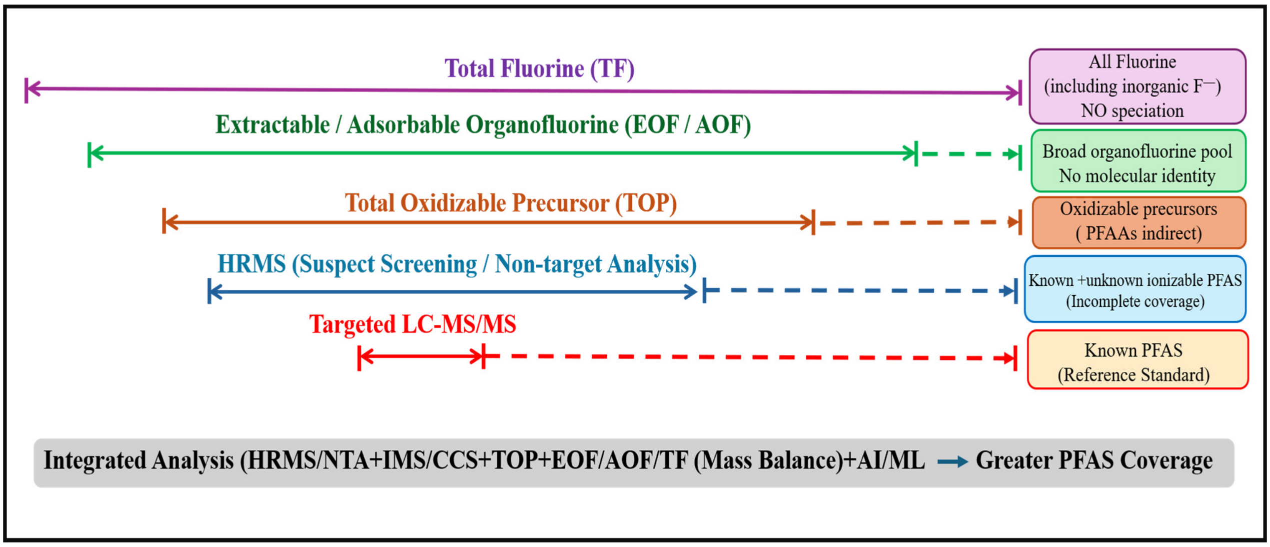

Figure S1. Conceptual comparison of the analytical coverage and remaining blind spots of complementary approaches for PFAS characterization. Dash lines indicate unresolved (analytical blind spots) analytical coverage.

Reference

1. Wang, B.; Yao, Y.; Sun, H.; Zhao, H.; Yu, Q. Contributions of Unknown Ionizable Precursors for Perfluoroalkyl Acids in the Air over a Wastewater Treatment Plant and a Landfill. *Environ. Geochem. Health* **2026**, *48*, 24, doi:10.1007/s10653-025-02856-4.

2. Liu, J.; Charbonnet, J.A. A Critical Review of PFAS Analysis, Occurrence, and Fate in Wastewater Treatment Plants. *Environ. Sci. Technol.* **2025**, *59*, 25492–25517, doi:10.1021/acs.est.5c09949.

3. Idowu, I.G.; Ekpe, O.D.; Megson, D.; Bruce-Vanderpuije, P.; Sandau, C.D. A Systematic Review of Methods for the Analysis of Total Per- and Polyfluoroalkyl Substances (PFAS). *Sci. Total Environ.* **2025**, *967*, 178644, doi:10.1016/j.scitotenv.2025.178644.

4. Leo WY Yeung, Hubert Dirven, Trine Husøy, Enmiao Jiao, Welmoed Nauta, Jana Geuer, Line Småstuen Haug, Dorte Herzke, M.A. Known and Unknown PFAS in Norwegian Adults: Temporal Trends and Sources. *Nord. Minist.* **2025**.

5. Huang, T.; Lyu, Y.; Fang, H.; Hu, J.; Li, T.; Zhao, Z.; Helbling, D.E.; Sun, W. Source-Specific Dynamics of Emerging Contaminants in a Megacity River Basin Revealed by Targeted and Nontargeted Analysis. *Environ. Sci. Technol.* **2026**, *60*, 19546–19557, doi:10.1021/acs.est.6c02605.

6. Jiao, E.; Zhu, Z.; Yin, D.; Qiu, Y.; Kärrman, A.; Yeung, L.W.Y. A Pilot Study on Extractable Organofluorine and Per- and Polyfluoroalkyl Substances (PFAS) in Water from Drinking Water Treatment Plants around Taihu Lake, China: What Is Missed by Target PFAS Analysis? *Environ. Sci. Process. Impacts* **2022**, *24*, 1060–1070, doi:10.1039/D2EM00073C.

7. McDonough, C.A.; Guelfo, J.L.; Higgins, C.P. Measuring Total PFASs in Water: The Tradeoff between Selectivity and Inclusivity. *Curr. Opin. Environ. Sci. Heal.* **2019**, *7*, 13–18, doi:10.1016/j.coesh.2018.08.005.

8. Liwara, D.J.; Baessmann, C.; Schneider, B.; Nordhorn, I.; Johansen, J.E.; Liu, H.; Koekkoek, J.; Heinzelmann, M.; de Boer, J.; Leonards, P.E.G.; et al. Comprehensive Per-and Polyfluoroalkyl Substances (PFAS) Screening in Dutch Surface Waters Using Target, Suspect, and Non-Targeted Approaches. *Environ. Pollut.* **2026**, *396*, 127846, doi:10.1016/j.envpol.2026.127846.

9. Nxumalo, T.; Akhdhar, A.; Mueller, V.; Simon, F.; von der Au, M.; Cossmer, A.; Pfeifer, J.; Krupp, E.M.; Meermann, B.; Kindness, A.; et al. EOF and Target PFAS Analysis in Surface Waters Affected by Sewage Treatment Effluents in Berlin, Germany. *Anal. Bioanal. Chem.* **2023**, *415*, 1195–1204, doi:10.1007/s00216-022-04500-x.

10. Lyu, X.; Xiao, F.; Shen, C.; Chen, J.; Park, C.M.; Sun, Y.; Flury, M.; Wang, D. Per- and Polyfluoroalkyl Substances (PFAS) in Subsurface Environments: Occurrence, Fate, Transport, and Research Prospect. *Rev. Geophys.* **2022**, *60*, doi:10.1029/2021RG000765.

11. Ruyle, B.J.; Pennoyer, E.H.; Vojta, S.; Becanova, J.; Islam, M.; Webster, T.F.; Heiger-Bernays, W.; Lohmann, R.; Westerhoff, P.; Schaefer, C.E.; et al. High Organofluorine Concentrations in Municipal Wastewater Affect Downstream Drinking Water Supplies for Millions of Americans. *Proc. Natl. Acad. Sci.* **2025**, *122*, doi:10.1073/pnas.2417156122.

12. Larsson, P.; Kärrman, A.; Yeung, L.W.Y. Elucidating Unknown Organofluorine in Municipal Wastewater: A Mass Balance Approach Including Fluorinated Pharmaceuticals. *Environ. Sci. Technol.* **2026**, *60*, 6623–6634, doi:10.1021/acs.est.5c13161.

13. Wang, Y.; Yu, N.; Zhu, X.; Guo, H.; Jiang, J.; Wang, X.; Shi, W.; Wu, J.; Yu, H.; Wei, S. Suspect and Nontarget Screening of Per- and Polyfluoroalkyl Substances in Wastewater from a Fluorochemical Manufacturing Park. *Environ. Sci. Technol.* **2018**, *52*, 11007–11016, doi:10.1021/acs.est.8b03030.

14. Herzke, D.; Nikiforov, V.; Yeung, L.W.Y.; Moe, B.; Routti, H.; Nygård, T.; Gabrielsen, G.W.; Hanssen, L. Targeted PFAS Analyses and Extractable Organofluorine – Enhancing Our Understanding of the Presence of Unknown PFAS in Norwegian Wildlife. *Environ. Int.* **2023**, *171*, 107640, doi:10.1016/j.envint.2022.107640.

15. Tokranov, A.K., Welch, S.M., Santangelo, L.M., Kent, D.B., Repert, D.A., Perkins, K., Bliznik, P.A., Roth, D.A., Drouin, A.F., Lincoln, T.A., Deyette, N.A., Schlosser, K.E.A., and Marts, J.M. Solid/Water Partitioning of Per- and Polyfluoroalkyl Substances (PFAS) in New Hampshire Soils and Biosolids: Results from Laboratory Experiments at the U.S. Geological Survey. *U.S. Geol. Surv.* **2023**, doi:https://doi.org/10.5066/P9TKSM8S.

16. Srivastava, P.; Macdonald, B. PFAS in Biosolids: Insights into Current and Future Challenges. *J. Hazard. Mater. Lett.* **2025**, *6*, doi:10.1016/j.hazl.2025.100163.

17. Spaan, K.M.; van Noordenburg, C.; Plassmann, M.M.; Schultes, L.; Shaw, S.; Berger, M.; Heide-Jørgensen, M.P.; Rosing-Asvid, A.; Granquist, S.M.; Dietz, R.; et al. Fluorine Mass Balance and Suspect Screening in Marine Mammals from the Northern Hemisphere. *Environ. Sci. Technol.* **2020**, *54*, 4046–4058, doi:10.1021/acs.est.9b06773.

18. Lauria, M.Z.; Sepman, H.; Ledbetter, T.; Plassmann, M.; Roos, A.M.; Simon, M.; Benskin, J.P.; Kruve, A. Closing the Organofluorine Mass Balance in Marine Mammals Using Suspect Screening and Machine Learning-Based Quantification. *Environ. Sci. Technol.* **2024**, *58*, 2458–2467, doi:10.1021/acs.est.3c07220.

19. Haque, F.; Soerensen, A.L.; Sköld, M.; Awad, R.; Spaan, K.M.; Lauria, M.Z.;

Plassmann, M.M.; Benskin, J.P. Per- and Polyfluoroalkyl Substances (PFAS) in White-Tailed Sea Eagle Eggs from Sweden: Temporal Trends (1969–2021), Spatial Variations, Fluorine Mass Balance, and Suspect Screening. *Environ. Sci. Process. Impacts* **2023**, *25*, 1549–1563, doi:10.1039/D3EM00141E.

20. Yeung, L.W.Y.; Mabury, S.A. Are Humans Exposed to Increasing Amounts of Unidentified Organofluorine? *Environ. Chem.* **2016**, *13*, 102–110, doi:10.1071/EN15041.

21. Cioni, L.; Nikiforov, V.; Benskin, J.P.; Coêlho, A.C.M.F.; Dudášová, S.; Lauria, M.Z.; Lechtenfeld, O.J.; Plassmann, M.M.; Reemtsma, T.; Sandanger, T.M.; et al. Combining Advanced Analytical Methodologies to Uncover Suspect PFAS and Fluorinated Pharmaceutical Contributions to Extractable Organic Fluorine in Human Serum (Tromsø Study). *Environ. Sci. Technol.* **2024**, *58*, 12943–12953, doi:10.1021/acs.est.4c03758.

22. Salangad, O.; Bouchikhi, A.; Cappelli, F.; Simon, F.; Buytaert, J.; Groffen, T.; Bervoets, L.; Covaci, A. Integrating Direct Total Oxidizable Precursor (DTOP) Assay with Suspect and Non-Target Screening Methods for Analysis of PFAS in Highly Contaminated Soils. *Emerg. Contam.* **2026**, 100718, doi:10.1016/j.emcon.2026.100718.

23. Kudzin, G.P.; Dodds, J.N.; Baker, E.S. Uncovering PFAS and Halogenated Chemicals Using Nontargeted Analysis and the PFAS IMplementer for Mass Spectrometry (PIMMS). *Sci. Adv.* **2026**, *12*, doi:10.1126/sciadv.aeg0781.

24. Zhang, B.; Liu, J.; Qing, S.; Herath, T.M.; Zhao, H.; Klabklaydee, S.; Fu, Q.-L.; Kwon, E.; Takeuchi, N.; Wang, D.; et al. Accurate Detection and High Throughput Profiling of Unknown PFAS Transformation Products for Elucidating Degradation Pathways. *Water Res.* **2025**, *282*, 123645, doi:10.1016/j.watres.2025.123645.

25. Xiao, S.; Liu, T.; Hu, L.; Yang, B.; Ying, G. Non-Target and Target Screening and Risk Assessment of per - and Polyfluoroalkyl Substances in Textile Wastewater and Receiving River. *Sci. Total Environ.* **2024**, *927*, 171876, doi:10.1016/j.scitotenv.2024.171876.

26. Jiao, Z.; Taniyasu, S.; Yu, N.; Wang, X. Two-Layer Homolog Network Approach for PFAS Nontarget Screening and Retrospective Data Mining. *Nat. Commun.* **2025**, doi:10.1038/s41467-025-56035-1.

27. Chen, Y.; Liu, T.; Hu, L.; Chen, C.; Yang, B.; Ying, G. Unveiling Per- and Polyfluoroalkyl Substance Contamination in Chinese Paper Products and Assessing Their Exposure Risk. *Environ. Int.* **2024**, *185*, 108540,

doi:10.1016/j.envint.2024.108540.

28. Schüßler, M.; Capitain, C.; Bugsel, B.; Zweigle, J.; Zwiener, C. Non - Target Screening Reveals 124 PFAS at an AFFF - Impacted Field Site in Germany Specified by Novel Systematic Terminology. *Anal. Bioanal. Chem.* **2025**, *417*, 6049–6064, doi:10.1007/s00216-024-05611-3.

29. Qiao, B.; Chen, H.; Song, D.; Fang, B.; Zhou, Y.; Yao, Y.; Sun, H. Nontarget Screening and Occurrence of Emerging Per- and Polyfluoroalkyl Substances in Municipal and Semiconductor Industrial Wastewater: A Large-Scale Survey in China. *Environ. Sci. Technol.* **2025**, *59*, 11829–11841, doi:10.1021/acs.est.5c02035.

30. Chen, Y.J.; Yang, J.S.; Yu, A.; Lin, C. Comprehensive Nontargeted Analysis of Fluorosurfactant Byproducts and Reaction Products in Wastewater from Semiconductor Manufacturing. *Sustain. Environ. Res.* **2024**, *1*, 1–16, doi:10.1186/s42834-024-00221-1.

31. Yao, J.; Sheng, N.; Guo, Y.; Yeung, L.W.Y.; Dai, J.; Pan, Y. Nontargeted Identification and Temporal Trends of Per- and Polyfluoroalkyl Substances in a Fluorochemical Industrial Zone and Adjacent Taihu Lake. *Environ. Sci. Technol.* **2022**, *56*, 7986–7996, doi:10.1021/acs.est.2c00891.

32. Zhang, B.; Meng, X.; Han, B.; Zhao, Z. Elaborating PFAS Characteristics for Chinese Electroplating Industry by Target and Nontarget Analysis. *Water Res.* **2025**, *287*, 124405, doi:10.1016/j.watres.2025.124405.

33. Li, M.; Hu, J.; Cao, X.; Chen, H.; Lyu, Y.; Sun, W. Nontarget Analysis Combined with TOP Assay Reveals a Significant Portion of Unknown PFAS Precursors in Firefighting Foams Currently Used in China. *Environ. Sci. Technol.* **2024**, *58*, 17104–17113, doi:10.1021/acs.est.4c07879.

34. Zhao, M.; Yao, Y.; Dong, X.; Baqar, M.; Fang, B.; Chen, H.; Sun, H. Nontarget Identification of Novel Per- and Polyfluoroalkyl Substances (PFAS) in Soils from an Oil Refinery in Southwestern China: A Combined Approach with TOP Assay. *Environ. Sci. Technol.* **2023**, *57*, 20194–20205, doi:10.1021/acs.est.3c05859.

35. Yao, Y.; Meng, Y.; Chen, H.; Zhu, L.; Sun, H. Non-Target Discovery of Emerging PFAS Homologues in Dagang Oilfield : Multimedia Distribution and Profiles in Crude Oil. *J. Hazard. Mater.* **2022**, *437*, 129300, doi:10.1016/j.jhazmat.2022.129300.

36. Li, W.; Jian, W.; Fu, Y. Basic Sample Preparation Techniques in LC-MS Bioanalysis. In *Sample Preparation in LC-MS Bioanalysis*; Wiley, 2019; pp. 1–30.

37. Lee, S.-H.; Tseng, W.-C.; Du, Z.-Y.; Lin, W.-Y.; Chen, M.-H.; Lin, C.-C.; Lien, G.-W.; Liang, H.-J.; Wen, H.-J.; Guo, Y.-L.; et al. Lipid Responses to Environmental

Perfluoroalkyl Substance Exposure in a Taiwanese Child Cohort. *Environ. Pollut.* **2021**, *283*, 117007, doi:10.1016/j.envpol.2021.117007.

38. Liu, Y.; Bao, J.; Hu, X.-M.; Lu, G.-L.; Yu, W.-J.; Meng, Z.-H. Optimization of Extraction Methods for the Analysis of PFOA and PFOS in the Salty Matrices during the Wastewater Treatment. *Microchem. J.* **2020**, *155*, 104673, doi:10.1016/j.microc.2020.104673.

39. Lockwood, T.E.; Talebi, M.; Minett, A.; Mills, S.; Doble, P.A.; Bishop, D.P. Micro Solid-Phase Extraction for the Analysis of per- and Polyfluoroalkyl Substances in Environmental Waters. *J. Chromatogr. A* **2019**, *1604*, 460495, doi:10.1016/j.chroma.2019.460495.

40. Pérez, R.A.; Albero, B. Ultrasound-Assisted Extraction Methods for the Determination of Organic Contaminants in Solid and Liquid Samples. *TrAC Trends Anal. Chem.* **2023**, *166*, 117204, doi:10.1016/j.trac.2023.117204.

41. Berger, U.; Haukås, M. Validation of a Screening Method Based on Liquid Chromatography Coupled to High-Resolution Mass Spectrometry for Analysis of Perfluoroalkylated Substances in Biota. *J. Chromatogr. A* **2005**, *1081*, 210–217, doi:10.1016/j.chroma.2005.05.064.

42. Liu, X.; Liu, Z.; Zhao, H.; Shi, R.; Wang, J.; Liu, J.; Di, S.; Wang, Z.; Wang, X.; Liu, L.; et al. Development and Validation of Modified QuEChERS Method Combined with LC-MS/MS for High-Throughput Analysis of per- and Polyfluoroalkyl Substances in Fish Samples. *Microchem. J.* **2024**, *199*, 110037, doi:10.1016/j.microc.2024.110037.

43. Camoiras González, P.; Sadia, M.; Baabish, A.; Sobhanei, S.; Fiedler, H. Air Monitoring with Passive Samplers for Perfluoroalkane Substances in Developing Countries (2017–2019). *Chemosphere* **2021**, *282*, 131069, doi:10.1016/j.chemosphere.2021.131069.

44. Olomukoro, A.A.; Emmons, R. V.; Godage, N.H.; Cudjoe, E.; Gionfriddo, E. Ion Exchange Solid Phase Microextraction Coupled to Liquid Chromatography/Laminar Flow Tandem Mass Spectrometry for the Determination of Perfluoroalkyl Substances in Water Samples. *J. Chromatogr. A* **2021**, *1651*, 462335, doi:10.1016/j.chroma.2021.462335.

45. Wang, R.; Wang, Y.; Hu, S.; Ding, X.; Zhang, H.; Hu, Z.; Liu, J.; Zhang, Q. A Novel Pretreatment Method for Fluorinated Alternatives in Soil and Sediment Using Accelerated Solvent Extraction. *npj Emerg. Contam.* **2025**, *1*, 13, doi:10.1038/s44454-025-00013-1.

46. Iannone, A.; Carriera, F.; Di Fiore, C.; Avino, P. Poly- and Perfluoroalkyl Substance (PFAS) Analysis in Environmental Matrices: An Overview of the Extraction and Chromatographic Detection Methods. *Analytica* **2024**, *5*, 187–202, doi:10.3390/analytica5020012.

47. Yanagisawa, H.; Obayashi, K.; Furuta, M.; Fujimaki, S. Screening of Adsorbable Per- and Polyfluoroalkyl Substances in Aqueous Environments Using Pyrolysis–GC–MS. *Sep. Sci. PLUS* **2026**, *9*, doi:10.1002/sscp.70191.

48. Forster, A.L.B.; Zhang, Y.; Westerman, D.C.; Richardson, S.D. Improved Total Organic Fluorine Methods for More Comprehensive Measurement of PFAS in Industrial Wastewater, River Water, and Air. *Water Res.* **2023**, *235*, 119859, doi:10.1016/j.watres.2023.119859.

49. von Abercron, E.; Falk, S.; Stahl, T.; Georgii, S.; Hamscher, G.; Brunn, H.; Schmitz, F. Determination of Adsorbable Organically Bound Fluorine (AOF) and Adsorbable Organically Bound Halogens as Sum Parameters in Aqueous Environmental Samples Using Combustion Ion Chromatography (CIC). *Sci. Total Environ.* **2019**, *673*, 384–391.

50. Miyake, Y.; Yamashita, N.; Rostkowski, P.; So, M.K.; Taniyasu, S.; Lam, P.K.S.; Kannan, K. Determination of Trace Levels of Total Fluorine in Water Using Combustion Ion Chromatography for Fluorine: A Mass Balance Approach to Determine Individual Perfluorinated Chemicals in Water. *J. Chromatogr. A* **2007**, *1143*, 98–104, doi:10.1016/j.chroma.2006.12.071.

51. Han, Y.; Pulikkal, V.F.; Sun, M. Comprehensive Validation of the Adsorbable Organic Fluorine Analysis and Performance Comparison of Current Methods for Total Per- and Polyfluoroalkyl Substances in Water Samples. *ACS ES&T Water* **2021**, *1*, 1474–1482, doi:10.1021/acsestwater.1c00047.

52. Wagner, A.; Raue, B.; Brauch, H.-J.; Worch, E.; Lange, F.T. Determination of Adsorbable Organic Fluorine from Aqueous Environmental Samples by Adsorption to Polystyrene-Divinylbenzene Based Activated Carbon and Combustion Ion Chromatography. *J. Chromatogr. A* **2013**, *1295*, 82–89.

53. Codling, G.; Vogt, A.; Jones, P.D.; Wang, T.; Wang, P.; Lu, Y.-L.; Corcoran, M.; Bonina, S.; Li, A.; Sturchio, N.C. Historical Trends of Inorganic and Organic Fluorine in Sediments of Lake Michigan. *Chemosphere* **2014**, *114*, 203–209.

54. Kaiser, A.-M.; Aro, R.; Kärrman, A.; Weiss, S.; Hartmann, C.; Uhl, M.; Forsthuber, M.; Gundacker, C.; Yeung, L.W.Y. Comparison of Extraction Methods for Per-and Polyfluoroalkyl Substances (PFAS) in Human Serum and Placenta Samples—Insights into Extractable Organic Fluorine (EOF) Kaiser A.-M. et Al. *Anal. Bioanal.*

Chem. **2021**, *413*, 865–876.

55. Pan, Y.; Helbling, D.E. Revealing the Factors Resulting in Incomplete Recovery of Perfluoroalkyl Acids (PFAAs) When Implementing the Adsorbable and Extractable Organic Fluorine Methods. *Water Res.* **2023**, *244*, 120497, doi:10.1016/j.watres.2023.120497.
